# Interplay between vaccines and treatment for dengue control: An epidemic model

**DOI:** 10.1371/journal.pone.0295025

**Published:** 2024-01-25

**Authors:** Abdoulaye Sow, Cherif Diallo, Hocine Cherifi

**Affiliations:** 1 Department of Computer Science, Algebra Laboratory Cryptography Codes and Applications, Gaston Berger University, Sanit-Louis, Senegal; 2 Department of Computer Science, LIB Burgundy Computer Laboratory -EA 7534, University of Burgundy Franche-Comte, Dijon, France; Parahyangan Catholic University: Universitas Katolik Parahyangan, INDONESIA

## Abstract

Assessing public health intervention strategies is crucial for effectively managing dengue. While numerous studies have explored the impact of dengue interventions on its transmission dynamics, limited research has focused on the combined effects of implementing multiple therapeutic interventions for disease control. This study presents an epidemic model for understanding dengue transmission dynamics, incorporating two critical therapeutic measures: vaccination and treatment of infected individuals. The model is characterized by ordinary differential equations involving seven-state variables. The investigation encompasses both disease-free and endemic equilibria of the model. The findings reveal that the disease-free equilibrium (only) is globally stable when the basic reproduction number is below one. Interestingly, when the vaccine’s effectiveness is low, treatment emerges as a more successful approach in reducing dengue cases than vaccination. In contrast, a highly effective vaccine alone significantly curtails dengue occurrences. Moreover, the study introduces an optimal control problem, featuring an objective function integrating two control mechanisms: vaccination and treatment. The analysis strongly suggests that implementing two control strategies outweighs the efficacy of a single approach in effectively mitigating the spread of the disease.

## Introduction

Dengue fever, caused by a virus prevalent in tropical and subtropical regions globally, presents a significant health concern. Annually, the World Health Organization (WHO) records over 4 million cases of dengue fever, with nearly 500,000 individuals suffering from its severe manifestation [1]. In recent years, authorities have implemented various non-pharmaceutical interventions, including vaccine research, controlling vector mosquitoes in affected areas, and promoting personal protection against mosquito bites. While no specific treatment for dengue fever exists, providing supportive care is crucial for preventing complications and potential fatalities. In severe dengue cases, skilled medical attention by professionals familiar with the disease’s effects and progression can drastically reduce the mortality rate from over 20% to less than 1% [2]. A dengue vaccine is available, administered in three doses spread over six months to individuals aged 9 to 45 [3]. However, recent research indicates potential long-term safety concerns among vaccinated individuals lacking prior exposure to the virus.

Given the virus’s ability to infect millions, dengue fever incurs substantial economic costs. Ongoing scientific efforts focus on devising effective intervention strategies to reduce dengue infections and curb the rapid spread of the disease. Consequently, researchers emphasize the need for practical assessments of public health intervention strategies, which are crucial for disease control and informing intervention policies.

Researchers have proposed various models to define disease transmission dynamics and evaluate the evolution or severity of dengue.

Esteva and Vargas [4] were among the first to study dengue transmission. They developed a mathematical model with compartmental models for human and mosquito populations. A susceptible-infected-recovered (SIR) model describes the human population, while an (SI) model describes the mosquito population. Singh et al. [5] and Tasman et al. [6] consider the effects of vaccination on a model dividing the human population into children and adults. They also assume two types of infection, primary and secondary, where people suffering from a secondary condition run a higher risk. Sow et al. [7] build a transmission model for a disease similar to dengue, zika, but considering vector-borne and human-to-human transmission in the disease’s transmission dynamics. Meksianis Z. Ndii et al. [8] use a deterministic dynamic model to study the interaction between media publicity of vaccination and disease seasonality and progression. They show that the waning immunity rate is one of the influential factors contributing to the increase in dengue infections, indicating the possibility of a higher number of secondary infections. Anusit Chamnan [9] determines the optimal control when only individuals with a documented history of dengue infection are vaccinated.

Sylvestre Aureliano Carvalho et al. [10] examine the efficacy of vaccination by transferring a proportion of individuals, relative to the vaccination rate, from the susceptible group to the recovered group. Their findings revealed that complete eradication of the dengue epidemic necessitates the implementation of an immunizing vaccine. Vector-targeted control measures alone are inadequate in halting the disease’s propagation. Indeed, when removing all infected mosquitoes from the system, susceptible mosquitoes persist. Consequently, human infections contribute to the resurgence of dengue within the human population through the remaining vector population. Ananya Dwivedi et al. [11] investigate the transmission dynamics of the dengue virus using a nonlinear vector-host model. The model considers both vaccination and treatment as control measures. Their research indicates that optimal treatment strategies significantly decrease hospitalizations and the number of infected individuals. MZ Ndii et al. [12] constructed deterministic and stochastic dengue epidemic models involving two age groups. Their findings indicate that vaccinating adult individuals results in fewer instances of infection among adults, offering population-level insights into the potential benefits of dengue vaccination. E Shim [13] develops a model considering antibody-dependent enhancement to find the best vaccination strategy. His findings emphasize the potential benefits of dengue vaccination despite limited vaccine efficacy. Ferguson et al. [14] assess the advantages and risks associated with dengue vaccine usage. They found an elevated risk of hospitalization when vaccination is introduced in regions with low to moderate transmission levels, while benefits emerge in areas with high transmission levels.

A recent review presents a comprehensive analysis of mathematical models for dengue epidemiology, encompassing multi-strain frameworks for transmission dynamics and within-host models [15]. By examining various models’ scope, approaches, and validation methods, the study provides insights for disease control strategies and enhances understanding of epidemiological and immunological factors influencing dengue transmission dynamics in real-world scenarios.

Although researchers have formulated numerous mathematical models to investigate dengue transmission dynamics in the context of vaccination, these models have primarily overlooked the potential synergies with treatment.

To the best of current knowledge, no prior work has formulated the issue in this specific manner. It is paramount to compare these interventions’ individual and combined performances rigorously. Regrettably, research exploring the combined impact of treatment and vaccination remains relatively scarce. Research investigating the ramifications of both vaccination and treatment on dengue fever underscores the potency of the dengue vaccine, which reduces disease severity by 88.5% and hospitalization by 67.2% [16].

However, there are other diseases for which vaccination and other forms of treatment have been studied jointly. One such example is the paper Modeling the Effects of Vaccination and Treatment on Pandemic Influenza, where Feng et al [17]. studied susceptible, infectious and cured models to assess the efficacy of various control programs using vaccination and antiviral treatment.

This article scrutinizes the effectiveness of vaccination and treatment in curbing dengue transmission. It showcases the outcomes of each strategy in isolation and synergy. To address this issue, we introduce a robust mathematical model. Unlike prior works, our analysis takes a unique angle by considering the intricate interplay between vaccination and treatment within the context of disease dynamics.

This novel perspective sheds light on the combined impact of these interventions and offers fresh insights into potential synergies that might have been overlooked.

By framing the issue this way, we aim to provide a more comprehensive understanding of the strategies’ effectiveness against dengue. This approach opens avenues for exploring hitherto unexplored dimensions of dengue control.

In the numerical analysis, we validated and estimated the parameters of the model under consideration by comparing model predictions with reported data on dengue infection in Kaohsiung, the main city in southern Taiwan and Thailand. To explore the robustness of the model to the parameter values used, we have studied sensitivity analysis using PRCC with Latin hypercube sampling. We are also numerically investigating the impact of vaccine efficacy and treatment rate on the epidemic growth rate.

The structure of the paper is as follows. Section 1 presents the proposed model’s general description and some dynamic behaviors. Section 2 gives the numerical simulations performed to support the theoretical results of the reference values for the model parameters. Section 3 identifies the influential model parameters that significantly affect the number of basic reproductions through a global sensitivity analysis. Sections 4 and 5 present the formulation and characterization of the control model and numerical simulations for two control strategies. Finally, we conclude with the discussion.

## Materials and methods

### Formulation of the mathematical model

The compartmental model has two parts: human compartments, denoted *h*, and vector compartments, subscripted with *v*. We use an *SVITR* (Susceptible, Vaccination, Infectious, Treatment, Recovered) model to describe the disease spread among the human population. Let *S*_*h*_(*t*), *V*_*h*_(*t*), *I*_*h*_(*t*), *T*_*h*_(*t*) and *R*_*h*_(*t*) denote the number of individuals in each compartment at time *t*. The human population at time *t* is given as

$$N_{h}\left(t\right)=S_{h}\left(t\right)+V_{h}\left(t\right)+I_{h}\left(t\right)+T_{h}\left(t\right)+R_{h}\left(t\right).$$

Moreover, the total mosquito population at time t, denoted by *N*_*v*_(*t*), is split into subpopulations of susceptible mosquitoes *S*_*v*_(*t*) and infected mosquitoes *I*_*v*_(*t*), thus

$$N_{v}\left(t\right)=S_{v}\left(t\right)+I_{v}\left(t\right).$$

**Fig 1:**
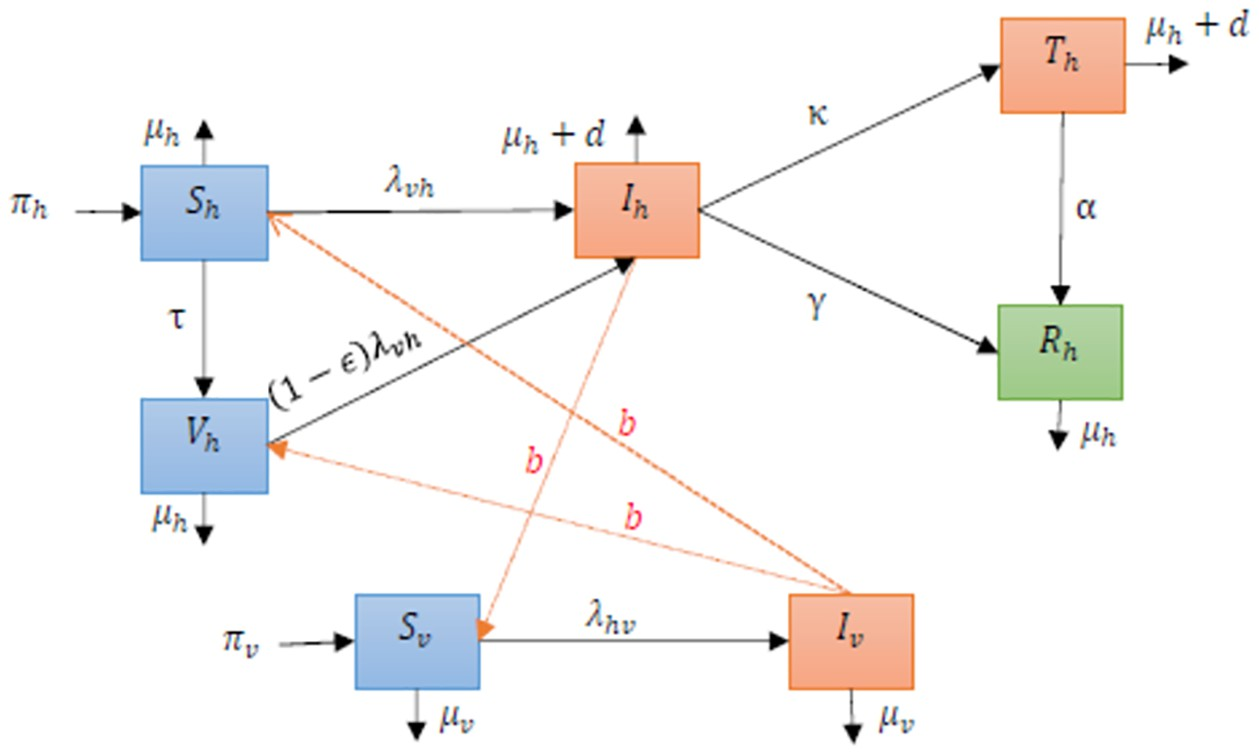
The flow chart represents the dynamical transmission in humans and mosquitoes, incorporating the effect of vaccination and treatment. The host population splits into states: Susceptible (*S*_*h*_), Vaccinated (*V*_*h*_), Infectious (*I*_*h*_), Treatment (*T*_*h*_) and Rcovered (*R*_*h*_). The vector population splits into 2 states: Susceptible (*S*_*v*_), and Infectious (*I*_*v*_). Dashed arrows show the direction of transmission between humans and mosquitoes.

We will use a single serotype dengue model that does not take into account the effects of secondary infections. Consequently, recovered individuals acquire lifelong immunity against the disease [18]. Transmission from susceptible to infectious state in human and vector populations is associated with mosquito bites *b*. Fig 1 depicts the schematic model flow. The model variables and parameters are described in Table 1. Since dengue vaccination is available, it is realistic to consider a specific vaccinated class *V*. The human population is vaccinated at a rate *τ*. The transmition rates from susceptible and vaccinated to infected is given by, respectively, by the following force of infection λ_*vh*_ and (1 − *ϵ*)λ_*vh*_ (*ϵ* represents the infection reduction of vaccinated individuals), thus

$$\begin{array}{c} \lambda_{vh}=\frac{b\beta_{vh}I_{h}}{N_{h}} \end{array}$$

**Table 1:** Values of parameters used in the model, and the sources used for the numerical values.

Parameter	Definition	Value	Source
*π*_*h*_	Recruitment rate of human	2.5	[19]
*b*	Mosquito biting rate	0.786	Estimated
*β*_*vh*_	Probability of mosquito infectiousness	0.84	Estimated
*β*_*hv*_	Probability of mosquito infection	0.78	Estimated
*γ*	Recovery rate for humans rate	0.2	[19]
*τ*	Vaccination rate	0.25	Assumed
*μ*_*h*_	Natural mortality rate of humans	0.000039	[19]
*ϵ*	Vaccine efficacy	0.538	[20]
*κ*	treatement rate of Infectious humans	0.12	[21]
*α*	Recovery rate of hospitalized individuals	0.4	[21]
*d*	Disease-induced mortality rate of humans	0.001	[19]
*π*_*v*_	Recruitment rate of vector	5000	[19]
*μ*_*v*_	Natural mortality rate of adult mosquitoes	0.17	Estimated

The transmition rate from susceptible mosquitoes to infected mosquitoes is given by,

$$\begin{array}{c} \lambda_{hv}=\frac{b\beta_{hv}I_{v}}{N_{h}} \end{array}$$

Infectious humans are treated at a rate *κ*, recover naturally at a rate *γ* and suffer disease-induced death at a rate *d*. Treated humans recover at a rate *α*. The features of transmission are illustrated in Fig 1, and the mathematical model is described by the following ordinary differential equations with the initial conditions

$$\begin{array}{c} \frac{dS_{h}}{dt}=\pi_{h}-\lambda_{vh}S_{h}-\left(\tau +\mu_{h}\right)S_{h}\\ \frac{dV_{h}}{dt}=\tau S_{h}-\left(1-\varepsilon \right)\lambda_{vh}V_{h}-\mu_{h}V_{h}\\ \frac{dI_{h}}{dt}=\lambda_{vh}S_{h}+\left(1-\varepsilon \right)\lambda_{vh}V_{h}-\left(\gamma +\kappa +d+\mu_{h}\right)I_{h}\\ \frac{dT_{h}}{dt}=\kappa I_{h}-\left(\alpha +d+\mu_{h}\right)T_{h}\\ \frac{dR_{h}}{dt}=\gamma \;I_{h}+\alpha T_{h}-\mu_{h}R_{h}\\ \frac{dS_{v}}{dt}=\pi_{v}-\lambda_{hv}S_{v}-\mu_{v}S_{v}\\ \frac{dI_{v}}{dt}=\lambda_{hv}S_{v}-\mu_{v}I_{v} \end{array}$$ (1)

$$\begin{array}{c} S_{h}\left(0\right)\geq 0,V_{h}\left(0\right)\geq 0,I_{h}\left(0\right)\geq 0,T_{h}\left(0\right)\geq 0,R_{h}\left(0\right)\geq 0,S_{v}\left(0\right)\geq 0,I_{v}\left(0\right)\geq 0 \end{array}$$ (2)

### Properties of solutions

The model proposed (1) is a system of non-linear ordinary differential equations with initial conditions (2). To be meaningful from an epidemiological and mathematical point of view, all solutions with the given initial conditions must remain non-negative and bounded for all finite time. The model must be analyzed in a biologically meaningful realizable region defined by a positive invariant set.

**Lemma 1** *Let F*(*t*) = (*S*_*h*_, *V*_*h*_, *I*_*h*_, *T*_*h*_, *R*_*h*_, *S*_*v*_, *I*_*v*_) *such that F*(0) ≥ 0. *Then the solutions F*(*t*) *of the model* (1) *are non negative for all* *t* ≥ 0.

**Proof 1** *Let T* = sup{*t* > 0 : *F*(*t*) > 0}. *In model* (1) *we have*:

$$\frac{dS_{h}}{dt}=\pi_{h}-\lambda_{vh}S_{h}\left(t\right)-\left(\tau +\mu_{h}\right)S_{h}\left(t\right)$$

*with* $\lambda_{vh}=\frac{b\beta_{vh}I_{v}}{N_{h}}$ *we can write the* λ_*vh*_ *as a function of* *I*_*v*_ *and N*_*h*_. *This gives*:

$$\frac{dS_{h}}{dt}=\pi_{h}-\lambda_{vh}\left(I_{v},N_{h}\right)S_{h}\left(t\right)-\left(\tau +\mu_{h}\right)S_{h}\left(t\right)$$

*By integration we obtain*

$$\frac{d}{dt}\{S_{h}\left(t\right)[\text{exp}\;(\int_{0}^{T}\lambda_{vh}\left(I_{v}\left(x\right),N_{h}\left(x\right)\right)dx)+\left(\tau +\mu_{h}\right)T]\}$$

$$=\pi \;\text{exp}\;(\int_{0}^{T}\lambda_{vh}\left(I_{v}\left(x\right),N_{h}\left(x\right)\right)dx+\left(\tau +\mu_{h}\right)T)$$

*Therefore*

$$S_{h}\left(T\right)\;\text{exp}\;[(\int_{0}^{T}\lambda_{vh}\left(I_{v}\left(x\right),N_{h}\left(x\right)\right)dx+\left(\tau +\mu_{h}\right)T)]-S_{h}\left(0\right)$$

$$=\int_{0}^{T}\pi \;\text{exp}\;(\int_{0}^{y}\lambda_{vh}\left(I_{v}\left(x\right),N_{h}\left(x\right)\right)dx+\left(\tau +\mu_{h}\right)y)dy$$

*Or*

$$S_{h}\left(T\right)=S_{h}\left(0\right)\;\text{exp}\;[-(\int_{0}^{T}\lambda_{vh}\left(I_{v}\left(x\right),N_{h}\left(x\right)\right)dx+\left(\tau +\mu_{h}\right)T)]$$

$$+exp[-(\int_{0}^{T}\lambda_{vh}\left(I_{v}\left(x\right),N_{h}\left(x\right)\right)dx+\left(\tau +\mu_{h}\right)T)]$$

$$\times \int_{0}^{T}\pi exp(\int_{0}^{y}\lambda_{vh}\left(I_{v}\left(x\right),N_{h}\left(x\right)\right)dx+\left(\tau +\mu_{h}\right)y)dy>0$$

*Similarly, we can prove that V*_*h*_(*T*), *I*_*h*_(*T*), *T*_*h*_(*T*), *R*_*h*_(*T*), *S*_*v*_(*T*) *and I*_*v*_(*t*) *all are psitive for all T* > 0 *therefore F* > 0 *for all t* > 0

**Theorem 2** *The closed region* $\Omega =\{\left(S_{h},V_{h},I_{h},T_{h},R_{h},S_{v},I_{v}\right)\in R_{+}^{7},N_{h}\leq \frac{\pi_{h}}{\mu_{h}},N_{v}\leq \frac{\pi_{v}}{\mu_{v}}\}$ *is a positively invariant set for the model* (1) *with non-negative initial condition in* $R_{+}^{7}$

**Proof 2** *here we use the basic theory of dynamical systems as described in* [22]

*As the total population sizes are*

$$N_{h}\left(t\right)=S_{h}\left(t\right)+V_{h}\left(t\right)+I_{h}\left(t\right)+T_{h}\left(t\right)+R_{h}\left(t\right)\geq 0\;\forall t>0$$

*and*

$$N_{v}\left(t\right)=S_{v}\left(t\right)+I_{v}\left(t\right)\geq 0\;\forall t>0$$

*we get*

$$\frac{dN_{h}}{dt}=\pi_{h}-\mu_{h}N_{h}-d\left(I_{h}+T_{h}\right)\;\text{and}\;\frac{dN_{v}}{dt}=\pi_{v}-\mu_{v}N_{v}$$

$$\frac{dN_{h}}{dt}\leq \pi_{h}-\mu_{h}N_{h}$$

$$\frac{dN_{v}}{dt}\leq \pi_{v}-\mu_{v}N_{v}$$

$$N_{h}\leq N_{h}\left(0\right)e^{-\mu_{h}\left(t\right)}+\frac{\pi_{h}}{\mu_{h}}(1-e^{-\mu_{h}\left(t\right)})$$

$$N_{v}\leq N_{v}\left(0\right)e^{-\mu_{v}\left(t\right)}+\frac{\pi_{v}}{\mu_{v}}(1-e^{-\mu_{v}\left(t\right)})$$

*which shows that*

$$\underset{t\to \infty }{\text{lim}\;\text{sup}}\;N_{h}\leq \frac{\pi_{h}}{\mu_{h}}$$

$$\underset{t\to \infty }{\text{lim}\;\text{sup}}\;N_{v}\leq \frac{\pi_{v}}{\mu_{v}}$$

*The initial conditions given* (2) *ensure that* *N*_*h*_(0) ≥ 0 *and N*_*v*_(0) ≥ 0. *Thus* *N*_*h*_(*t*) *and N*_*v*_(*t*) *are positively-bounded for all* *t* ≥ 0

### Disease-free equilibrium and basic reproduction number

The basic reproduction ratio, *R*_0_, gives the average number of secondary cases of infection resulting from a single primary infection in a population where everyone is susceptible. In epidemic models, it is worthy of consideration as it indicates the persistence or eradication of diseases by giving a threshold depending on epidemiological parameters. Indeed, an epidemic is said to be under control (cannot persist in the population) when *R*_0_ < 1.

The disease-free equilibrium (DFE) of the model (1) is obtained by using *I*_*h*_ = 0, *T*_*h*_ = 0 and *I*_*v*_ = 0 in the steady state conditions:

$$\begin{array}{c} E_{0}=(\frac{\pi_{h}}{\tau +\mu_{h}},\frac{\tau \pi_{h}}{\mu_{h}\left(\tau +\mu_{h}\right)},0,0,0,0,\frac{\pi_{v}}{\mu_{v}}) \end{array}$$

We use the following generation operator method on model (1) to calculate the basic reproduction number.

Consider the infected compartments *I*_*h*_; *T*_*h*_; *I*_*v*_ at the disease-free equilibrium (DFE) and applying [23] technique, the Jacobian matrices *F* and *V* for the new infection terms and the remaining transfer terms respectively are given by

$$\begin{array}{c} F=(\begin{array}{ccc} 0 & 0 & \frac{b\beta_{vh}S_{h}^{0}+b\left(1-\varepsilon \right)\beta_{vh}V_{h}^{0}}{N_{h}^{0}}\\ 0 & 0 & 0\\ \frac{b\beta_{hv}S_{v}^{0}}{N_{h}^{0}} & 0 & 0 \end{array})\\ V=(\begin{array}{ccc} \gamma +\kappa +d_{h}+\mu_{h} & 0 & 0\\ -\kappa & \alpha +\mu_{h}+d_{h} & 0\\ 0 & 0 & \mu_{v} \end{array}) \end{array}$$

Thus, the effective reproduction number is given by

$$\begin{array}{c} R_{0}=\sqrt{\frac{b^{2}\beta_{vh}\beta_{hv}\mu_{h}^{2}\pi_{v}}{\pi_{h}\mu_{v}^{2}\left(\tau +\mu_{h}\right)\left(\gamma +\kappa +d+\mu_{h}\right)}[1+\frac{\tau (1-\varepsilon )}{\mu_{h}}]} \end{array}$$ (3)

### Global asymptotic stability of the disease-free equilibrium

In this section, we study the global asymptotic stability of the disease-free equilibrium (DFE) to assure the eradication of the Dengue virus. We consider the feasible region

$$\Gamma =\{\left(S_{h},V_{h},I_{h},T_{h},R_{h},S_{v},I_{v}\right)\in R_{+}^{7},S_{h}\leq S_{h}^{0},S_{v}\leq S_{v}^{0}\}$$

**Lemma 3** Γ *is a positively invariant set for the model* (1) *with non- negative initial condition in* $R^{7}$

**Proof 3** *From model* (1) *we have*

$$\frac{dS_{h}}{dt}\left(t\right)=\pi_{h}-\lambda_{vh}S_{h}\left(t\right)-\left(\mu_{h}+\tau \right)S_{h}\left(t\right)\leq \pi_{h}-\left(\mu_{h}+\tau \right)S_{h}\left(t\right)=\left(\mu_{h}+\tau \right)(\frac{\pi_{h}}{\left(\mu_{h}+\tau \right)}-S_{h}\left(t\right))$$

*and we know that* $S_{h}\left(0\right)=\frac{\pi_{h}}{\mu +\tau }$ *therefore* $S_{h}\left(t\right)\leq S_{h}\left(0\right)-\left(S_{h}^{0}-S_{h}\left(0\right)\right)e^{-(\mu_{h}+\tau )t}$ .

*So, if* $S_{h}\left(0\right)\leq S_{h}^{0}\forall t\geq 0S_{h}\left(t\right)\leq S_{h}^{0}\;\forall t\geq 0$ . *and also*

$$\frac{dS_{v}}{dt}\left(t\right)=\pi_{v}-\lambda_{vh}S_{v}\left(t\right)-\mu_{v}S_{v}\left(t\right)\leq \pi_{v}-\mu_{v}S_{v}\left(t\right)=\mu_{v}(\frac{\pi_{v}}{\mu_{v}}-S_{v}\left(t\right))=\mu_{v}\left(S_{v}\left(0\right)-S_{v}\left(t\right)\right)$$

$S_{v}\left(0\right)=\frac{\pi_{v}}{\mu_{v}}S_{v}\left(t\right)\leq S_{v}\left(0\right)-\left(S_{v}^{0}-S_{v}\left(0\right)\right)e^{-\mu_{v}t}$. *Thus if* $S_{v}\left(0\right)\leq S_{v}^{0}\;\forall t\geq 0S_{v}\left(t\right)\leq S_{v}^{0}\;\forall t\geq 0$

**Lemma 4** *Let* $\left(X_{1}^{0},\vec{0}\right)$ *such that* $X_{1}^{0}$ *is a globally stable equilibrium. If* *G*(*X*_1_, *X*_2_) *satisfies the following two conditions given in* [24], *namely* $G\left(X_{1},\vec{0}\right)=\vec{0}\;and\;\hat{G}\left(X_{1},X_{2}\right)=D_{X_{2}}G\left(X_{1}^{0},\vec{0}\right)X_{2}-G\left(X_{1},X_{2}\right),\vec{G}\left(X_{1},X_{2}\right)\geq 0$, *then the disease free equilibrium is globally asymptotically stable*

**Theorem 5** *(global asymptotic stability of the DFE). The DFE* Γ *of model* (1) *is globally asymptotically stable if R*_0_ < 1:

**Proof 4** *Let*

$$X_{1}=\left(S_{h},V_{h},R_{h},S_{v}\right),\;X_{2}=\left(I_{h},T_{h},I_{v}\right)$$

*and by grouping model* (1) *into*

$$\frac{dX_{1}}{dt}=F\left(X_{1},0\right),\frac{dX_{2}}{dt}=G\left(X_{1},X_{2}\right)$$

*where F*(*X*_1_, 0) *is obtained from the right-hand side of the first, second, fifth and sixth equations of model* (1) *with* *I*_*h*_ = *T*_*h*_ = *Iv* = 0 *and* *G*(*X*_1_;*X*_2_) *is obtained from the right-hand side of the third, fourth and seventh equations of model* (1).

*Now consider the model in reduced form*:

$$\begin{array}{c} \frac{dS_{h}}{dt}=\pi_{h}-\left(\tau +\mu_{h}\right)S_{h}\\ \frac{dV_{h}}{dt}=\tau S_{h}-\mu_{h}V_{h}\\ \frac{dR_{h}}{dt}=-\mu_{h}R_{h}\\ \frac{dS_{v}}{dt}=\pi_{v}-\mu_{v}S_{v} \end{array}$$ (4)

*This equation has a unique equilibrium point*

$$X_{1}^{0}=(\frac{\pi_{h}}{\tau +\mu_{h}},\frac{\tau \pi_{h}}{\mu_{h}\left(\tau +\mu_{h}\right)},0,\frac{\pi_{v}}{\mu_{v}})$$

*which is globally asymptotically stable*

*Next, we check G*(*X*_1_, *X*_2_) *satisfies two conditions of lemme 2 where*

$$\left(X_{1}^{0},\vec{0}\right)=(\frac{\pi_{h}}{\tau +\mu_{h}},\frac{\tau \pi_{h}}{\mu_{h}\left(\tau +\mu_{h}\right)},0,0,0,\frac{\pi_{v}}{\mu_{v}},0)$$

*and*

$$D_{X_{2}}G\left(X_{1}^{0},\vec{0}\right)=(\begin{array}{ccc} -\gamma -\kappa -d-\mu_{h} & 0 & \frac{b\beta_{vh}S_{h}+b\left(1-\varepsilon \right)\beta_{vh}V_{h}}{N_{h}^{0}}\\ k & -\alpha -d=\mu_{h} & 0\\ \frac{b\beta_{hv}S_{v}}{N_{h}^{0}} & 0 & -\mu_{v} \end{array})$$

*is the Jacobian of G*(*X*_1_, *X*_2_) *with respect to* (*I*_*h*_;*T*_*h*_;*I*_*v*_) *is calculated at* $\left(X_{1}^{0},\vec{0}\right)$ *It is an M-matrix with off-diagonal elements that are non-negative. The relation*

$$\hat{G}\left(X_{1},X_{2}\right)=D_{X_{2}}G\left(X_{1}^{0},\vec{0}\right)X_{2}-G\left(X_{1},X_{2}\right)$$

*gives*

$$\begin{array}{c} \hat{G}\left(X_{1},X_{2}\right)=(\begin{array}{c} \frac{b\beta_{vh}S_{v}^{0}}{N_{h}^{0}}\left(1-\frac{N_{h}^{0}S_{h}}{S_{h}^{0}N_{h}}\right)+\frac{b\beta_{vh}V_{h}^{0}\left(1-\epsilon \right)}{N_{h}^{0}}\left(1-\frac{N_{h}^{0}V_{h}}{V_{h}^{0}N_{h}}\right)\\ 0\\ \frac{b\beta_{hv}S_{v}^{0}}{N_{h}^{0}}\left(1-\frac{N_{h}^{0}S_{v}}{S_{v}^{0}N_{h}}\right) \end{array}) \end{array}$$

*In the region* Γ, *we have* $\hat{G}\left(X_{1},X_{2}\right)\geq 0$ *So by the theorem in* [24], *the global stability of the DFE is obtained*.

The above theorem indicates that the dengue virus can be eradicated from the population if *R*_0_ can be reduced to a value less than or equal to unity, regardless of the size of the initial sub-population in each class.

### Stability of the endemic equilibrium

The state of endemic equilibrium is the state in which the disease cannot be totally eradicated but persists in the population.

Let

$$E^{*}=\left(S_{h}^{*},V_{h}^{*},I_{h}^{*},T_{h}^{*},R_{h}^{*};S_{v}^{*},I_{v}^{*}\right)$$

After some algebraic manipulations, the endemic equilibrium of model system (3) is obtained as

$$S_{h}^{*}=\frac{\pi_{h}}{\lambda_{vh}^{*}+\left(\tau +\mu_{h}\right)},$$

$$V_{h}^{*}=\frac{\tau \pi_{h}}{\left(\lambda_{vh}^{*}+\left(\tau +\mu_{h}\right)\right)\left(\left(1-\varepsilon \right)+\mu_{h}\right)},$$

$$I_{h}^{*}=[\frac{\lambda_{vh}^{*}\pi_{h}}{\lambda_{vh}^{*}+\left(\tau +\mu_{h}\right)}+\frac{\left(1-\varepsilon \right)\tau \pi_{h}}{\left(\lambda_{vh}^{*}+\left(\tau +\mu_{h}\right)\right)\left(\left(1-\varepsilon \right)+\mu_{h}\right)}].\frac{1}{\left(\gamma +\kappa +d+\mu_{h}\right)},$$

$$S_{v}^{*}=\frac{\pi_{v}}{\lambda_{hv}^{*}+\mu_{v}},$$

$$I_{v}^{*}=\frac{\lambda_{hv}^{*}\pi_{v}}{\left(\lambda_{hv}^{*}+\mu_{v}\right)\mu_{v}}$$

with

$$\begin{array}{c} \lambda_{vh}^{*}=\frac{b\beta_{vh}I_{v}^{*}}{N_{h}}=\frac{b\beta_{vh}\cdot \frac{\lambda_{hv}^{*}\pi_{v}}{\left(\lambda_{hv}^{*}+\mu_{v}\right)\mu_{v}}}{N_{h}} \end{array}$$ (5)

and

$$\begin{array}{c} \lambda_{hv}^{*}=\frac{b\beta_{hv}I_{h}^{*}}{N_{h}}=\frac{b\beta_{hv}[\frac{\lambda_{vh}^{*}\pi_{h}}{\lambda_{vh}^{*}+\left(\tau +\mu_{h}\right)}+\frac{\left(1-\varepsilon \right)\tau \pi_{h}}{\left(\lambda_{vh}^{*}+\left(\tau +\mu_{h}\right)\right)\left(\left(1-\varepsilon \right)+\mu_{h}\right)}]}{N_{h}\left(\gamma +\kappa +d+\mu_{h}\right)} \end{array}$$ (6)

From the Eq (5) and using Eq (6) we obtain the following quadratic equation;

$$\begin{array}{c} \lambda_{hv}^{*}\left(c_{3}\lambda_{hv}^{*3}+c_{2}\lambda_{hv}^{*2}+c_{1}\lambda_{hv}^{*}+c_{0}\right)=0 \end{array}$$ (7)

If $\lambda_{hv}^{*}=0$, then *I*_*h*_ = 0, which represents the disease-free equilibrium. Otherwise

$$c_{3}\lambda_{hv}^{*3}+c_{2}\lambda_{hv}^{*2}+c_{1}\lambda_{hv}^{*}+c_{0}=0$$

Now by the Routh-Hurtwiz criteria, the eigenvalues of the endemic equilibrium state will have negative real parts if *c*_3_, *c*_2_ and *c*_1_ are positive constants and *c*_1_*c*_2_ > *c*_0_*c*_3_,

$$\begin{array}{c} c_{0}=\pi_{h}\left(\gamma +\kappa +d+\mu_{h}\right){\left(\tau +\mu_{h}\right)}^{2}\mu_{v}^{3}\left(R_{0}^{2}-1\right) \end{array}$$ (8)

and *c*_1_, *c*_2_ and *c*_3_ given in the appendix S1 Appendix. All these conditions will be satisfied if *R*_0_ > 1

**Theorem 6** The equilibrium point E* *is locally asymptotically stable when If* *R*_0_ > 1.

## Model fitting and parameter estimation

This section concerns parameter estimation (using least squares regression) of the dengue model based on weekly dengue case data from 2014–2015 in Kaohsiung, the main city in southern Taiwan [25]. In this paper, the values of parameters such as mosquito infection probability, human infection probability, mosquito bite rate and adult mosquito natural mortality rate are estimated, while the values of other parameters are chosen from the literature. The corresponding values of the estimated parameters are given in Table 1. In 2014–2015, the total population of Kaohsiung was estimated at 1768000 [26]. The initial total population is therefore *N*_*h*_(0) = 1768000. The initial number of infected populations is 10 and the initial density of the remaining populations is arbitrarily assumed Table 2.

**Table 2:** Summary table of initial state values.

Parameter	Definition	Value
*S*_*h*_(0)	initial susceptible human	1767970
*V*_*h*_(0)	initial vaccinated human	20
*I*_*h*_(0)	initial infected human	10
*T*_*h*_(0)	initial treatement human	0
*R*_*h*_(0)	initial recovered human	0
*S*_*v*_(0)	initial susceptible vector	3 × *S*_*h*_(0)
*I*_*v*_(0)	initial infected vector	100

**Fig 2:**
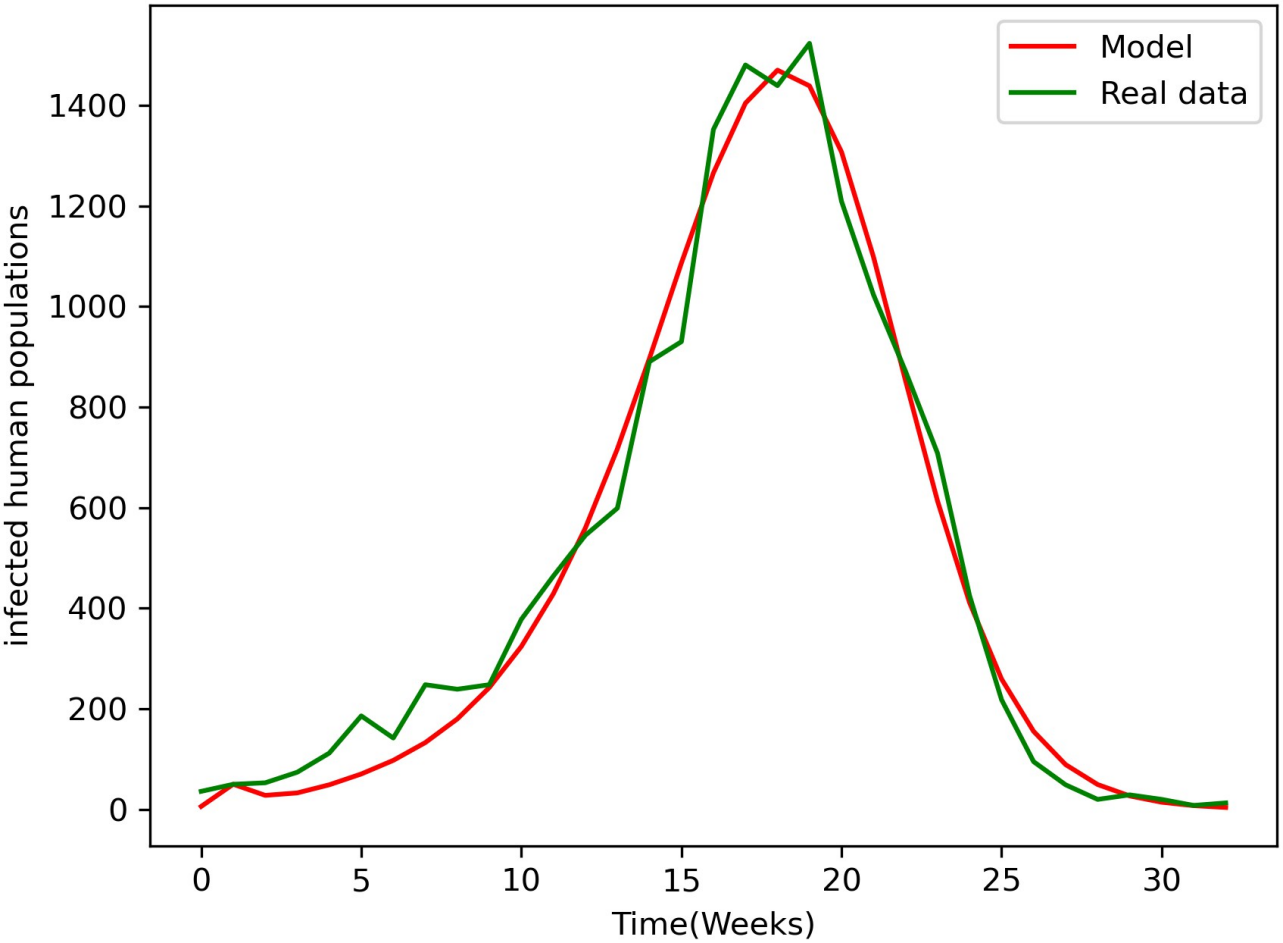
Best fit of models to reported dengue cases in Kaohsiung. The real data corresponds to the number of reported cases for the dengue outbreak occurred in Kaohsiung 2014–2015.

Fig 2 shows that the model captures the general behavior of dengue cases in this municipality.

## Numerical simulations

In this section, we conduct a numerical analysis of dengue transmission through vaccination and treatment using several distinct scenarios. The model’s parameter values are listed in Table 1.

**Fig 3:**
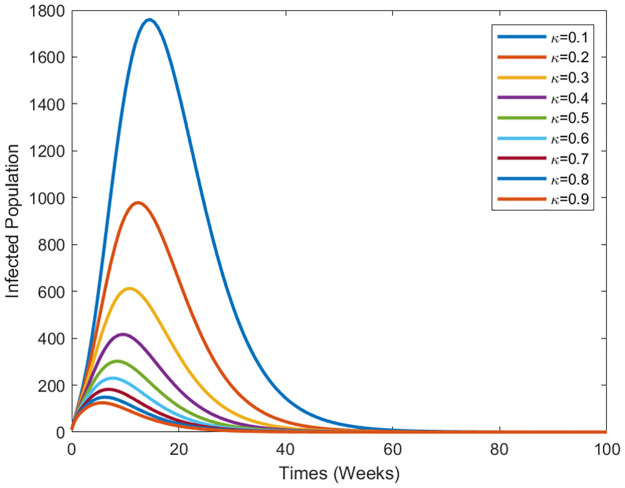
Evolution of the epidemic spreading in the human population for various values of the cure rate *κ*.

**Fig 4:**
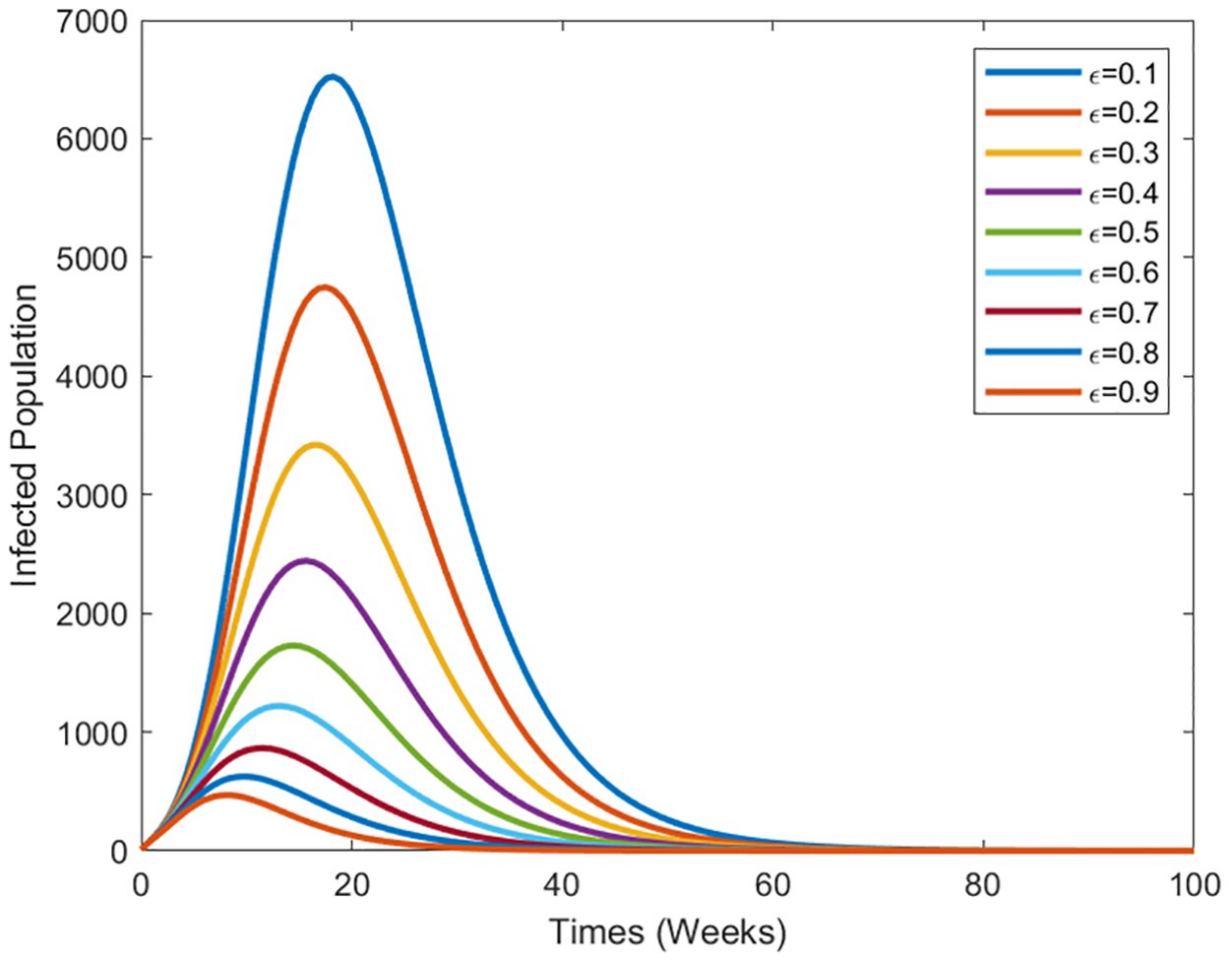
Evolution of the epidemic spreading in the human population for various values of the vaccine efficacy rate *ϵ*.

We perform numerical simulations to illustrate the dynamic behavior of the diseases. Fig 3 demonstrates the cure rate’s influence on the disease’s transmission dynamics. As the treatment strategy intensifies, the infected population diminishes. Fig 4 shows the effect of transmission of vaccination efficacy. the transmission effect of vaccination efficacy. As vaccination efficiency increases, the infected population decreases.

Here, we present the dengue reduction with three scenarios: vaccination, treatment, and both vaccination and treatment. We also show the numerical solutions of the model with different vaccination rates and vaccine efficacy.

**Fig 5:**
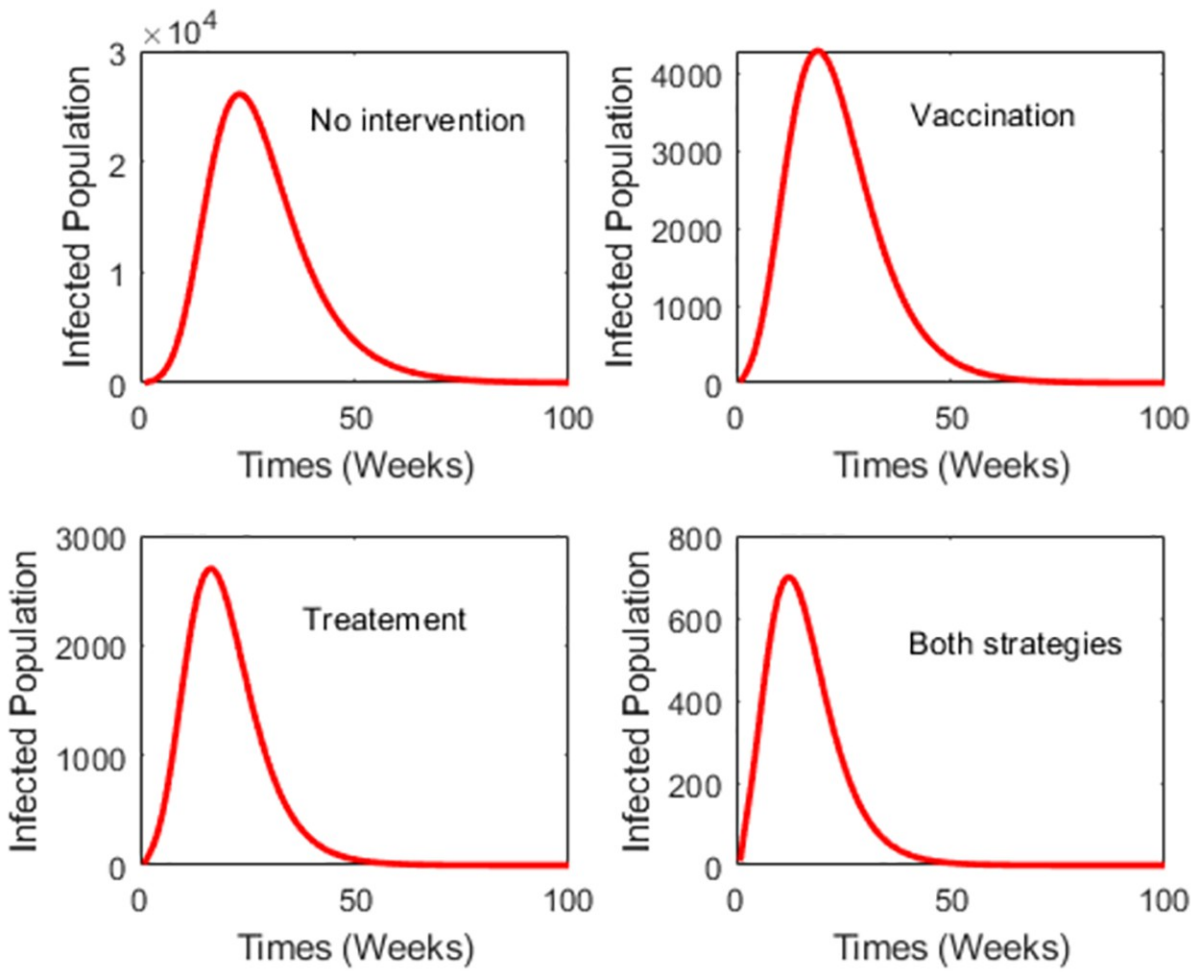
Numerical simulations of the model with no intervention, vaccine only, treatement only, and both vaccine and treatement. The vaccine efficacy is 0.536 and the vaccination rate is 0.2.

Fig 5 depicts the numerical solutions of the model with a vaccine efficacy of 0.536 and a vaccination rate of 0.2. Here, the vaccine efficacy of 0.536 reflects its effectiveness on seronegative individuals. The findings reveal that treatment alone significantly reduces dengue cases more than the vaccine. Specifically, using vaccination alone, treatment alone, and a combination of both strategies can curtail the number of dengue cases by approximately 74%, 89%, and 98%, respectively. It underscores that relying solely on treatment is sufficient to reduce dengue cases when the vaccine efficacy is low.

**Fig 6:**
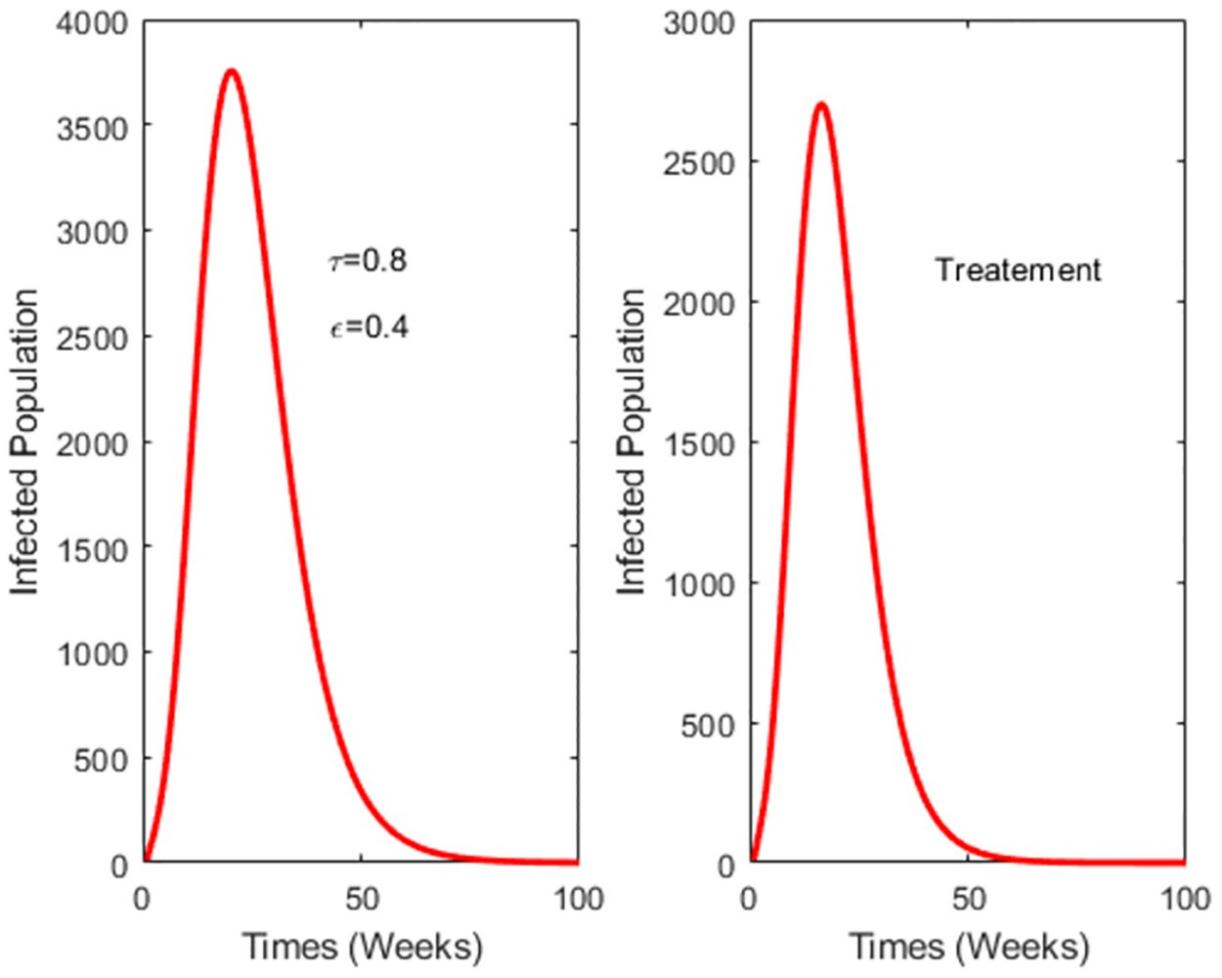
Comparing the performance of vaccination and treatment with high vaccination rates. *τ* = 0.8 and the vaccine efficacy is 0.4.

Fig 6 illustrates that despite a high vaccination rate, the effectiveness of treatment surpasses that of the vaccine. This outcome could potentially be influenced by a low vaccine efficacy, resulting in the reinfection of vaccinated individuals.

**Fig 7:**
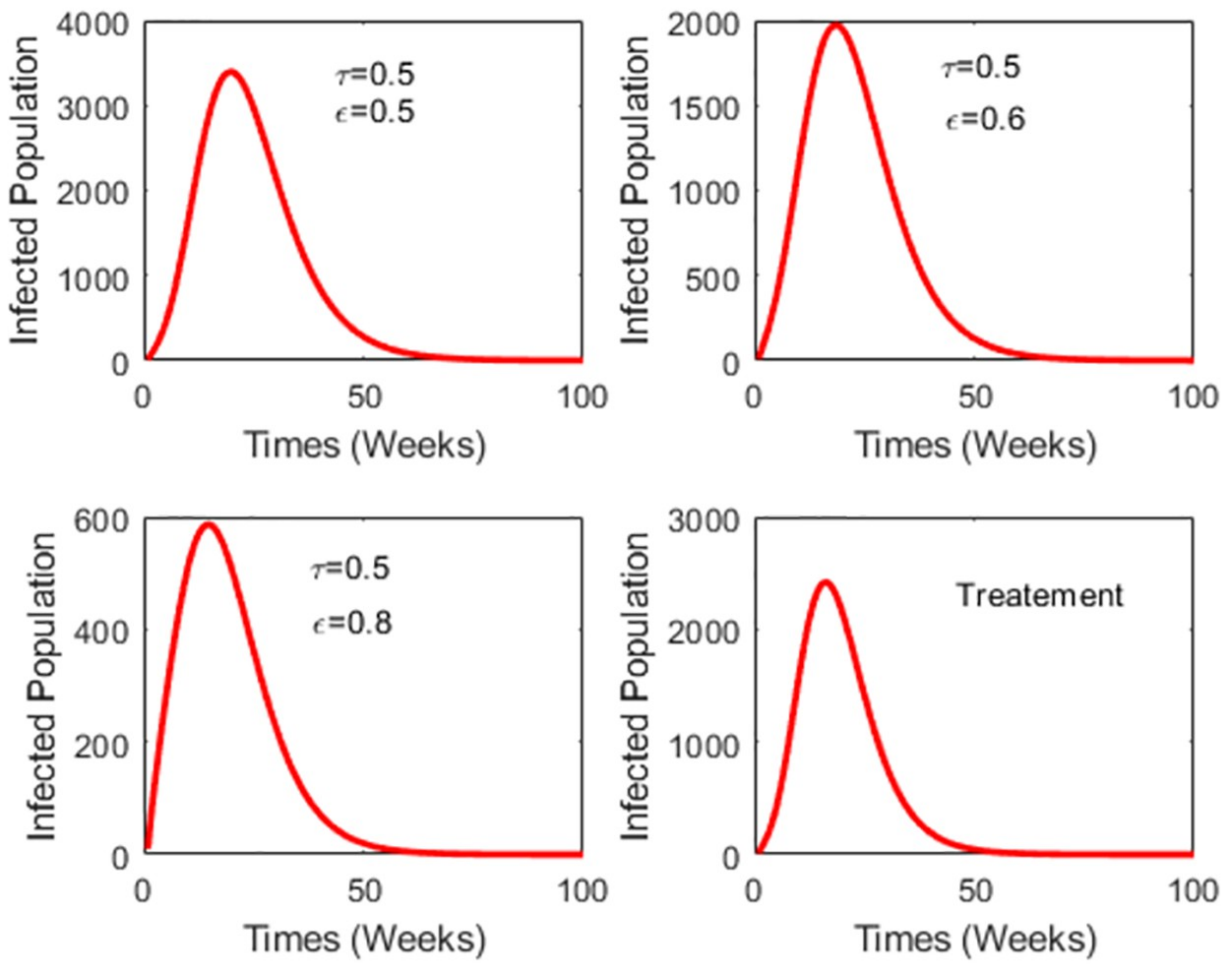
Comparing the performance of vaccination and treatment with different vaccine efficacy.

At a vaccination rate of 0.5 and vaccine efficacy of 0.8, Fig 7 demonstrates that the reduction in the number of dengue cases achieved through vaccination outweighs that achieved through treatment. It emphasizes the significance of factoring in vaccine efficacy and vaccination rate when devising a vaccination strategy.

### Vaccination campaign

We analyze the crucial vaccination coverage rate necessary for disease eradication. In the absence of vaccination within the community, the number of effective reproductions is:

$$\begin{array}{c} r_{0}=\sqrt{\frac{b^{2}\beta_{vh}\beta_{hv}\mu_{h}^{2}\pi_{v}}{\pi_{h}\mu_{v}^{2}\mu_{h}\left(\gamma +\kappa +d+\mu_{h}\right)}} \end{array}$$ (9)

We know

$$\begin{array}{c} R_{0}=\sqrt{\frac{b^{2}\beta_{vh}\beta_{hv}\mu_{h}^{2}\pi_{v}}{\pi_{h}\mu_{v}^{2}\left(\tau +\mu_{h}\right)\left(\gamma +\kappa +d+\mu_{h}\right)}[1+\frac{\tau (1-\varepsilon )}{\mu_{h}}]} \end{array}$$ (10)

After some rearrangement,

$$\frac{R_{0}^{2}}{r_{0}^{2}}=\frac{\mu_{h}}{\tau +\mu_{h}}+\frac{\tau }{\tau +\mu_{h}}\left(1-\epsilon \right)$$

$$\begin{array}{c} R_{0}=r_{0}\sqrt{\left[\frac{\mu_{h}}{\tau +\mu_{h}}+\frac{\tau }{\tau +\mu_{h}}\left(1-\epsilon \right)\right]} \end{array}$$ (11)

$$\begin{array}{c} R_{0}^{\infty }=\underset{\tau \to \infty }{\text{lim}}\;R_{0}=r_{0}\sqrt{1-\epsilon } \end{array}$$ (12)

$$\begin{array}{c} R_{0}\left(\infty \right)<1\Leftrightarrow r_{0}\sqrt{1-\epsilon }<1\Leftrightarrow \epsilon >1-\frac{1}{r_{0}^{2}} \end{array}$$ (13)

$$\epsilon^{*}=1-\frac{1}{r_{0}^{2}}\Leftrightarrow \epsilon >\epsilon^{*}$$

**Fig 8:**
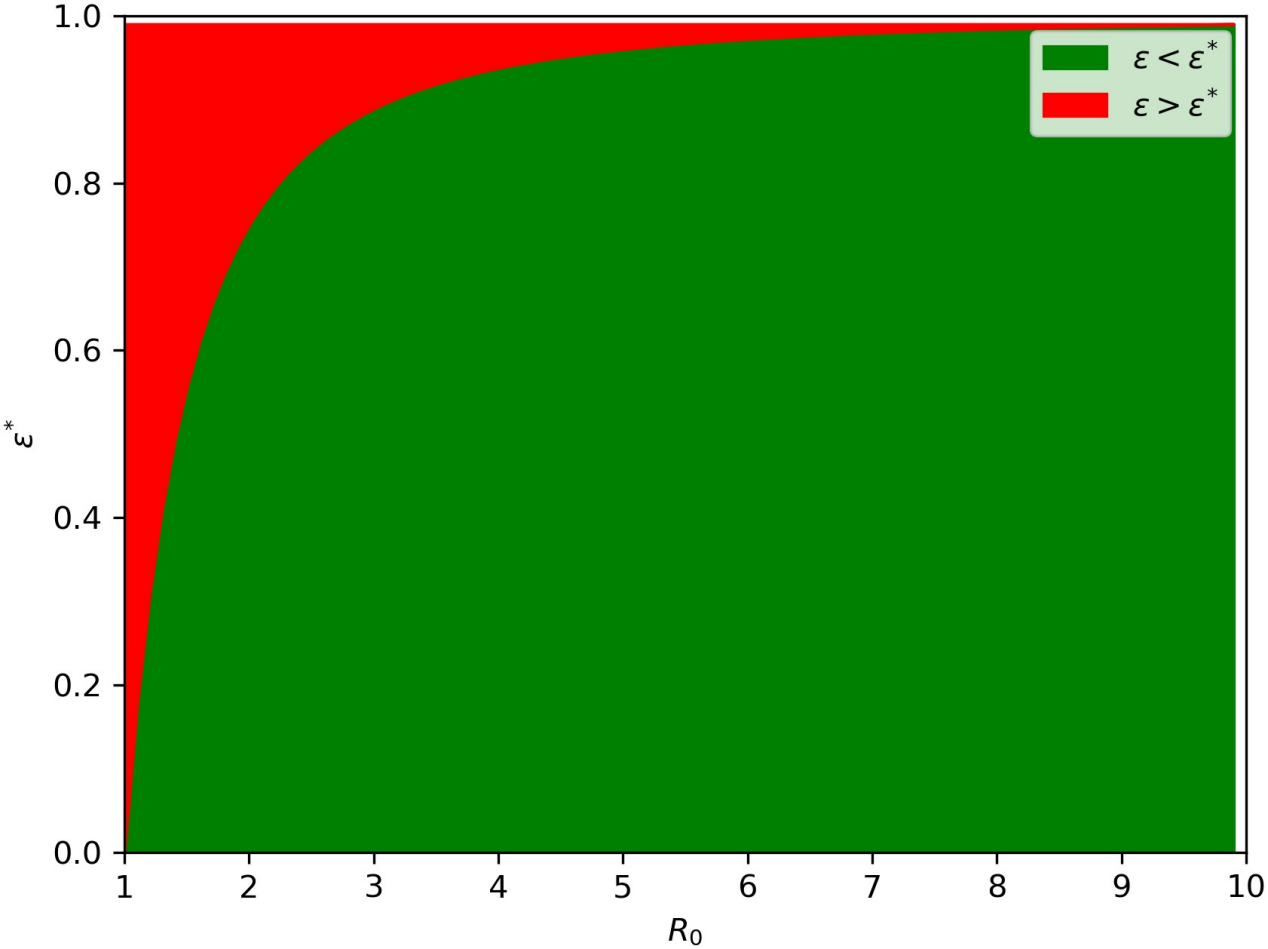
Theoretical impact of perfect vaccination. The graph shows the relationship between the critical level of vaccination required to eradicate infection and the basic reproduction rate, *R*_0_.

An average infected individual causing *R*_0_ secondary cases prompts us to consider immunizing at least $r_{0}^{2}-1$ of them to halt epidemic spread. It establishes a critical vaccination level for population protection. This value, derived from the differential equation model, represents the threshold against preventing a newly introduced infectious disease and eradicating an existing contagious one (see Fig 8). Notably, disease control does not require complete population vaccination. Indeed, each vaccinated individual contributes to collective immunity, reducing the likelihood of disease transmission to others they might have infected. Moreover, Fig 8 shows that additional efforts is necessary to reduce *R*_0_ below unity even when vaccination coverage *τ* is high.

## Global sensitivity analysis

The input parameters determine the results of the compartmental mathematical models, which may be subject to uncertainty. A global sensitivity analysis is therefore carried out to examine the effect of uncertainty and the sensitivity of numerical simulation results to changes in each parameter of our model. Therefore, a global sensitivity analysis is performed to determine the most influential model parameters using a combination of Latin Hypercube Sampling (LHS) and Partial Rank Coefficient (PRCC).

**Fig 9:**
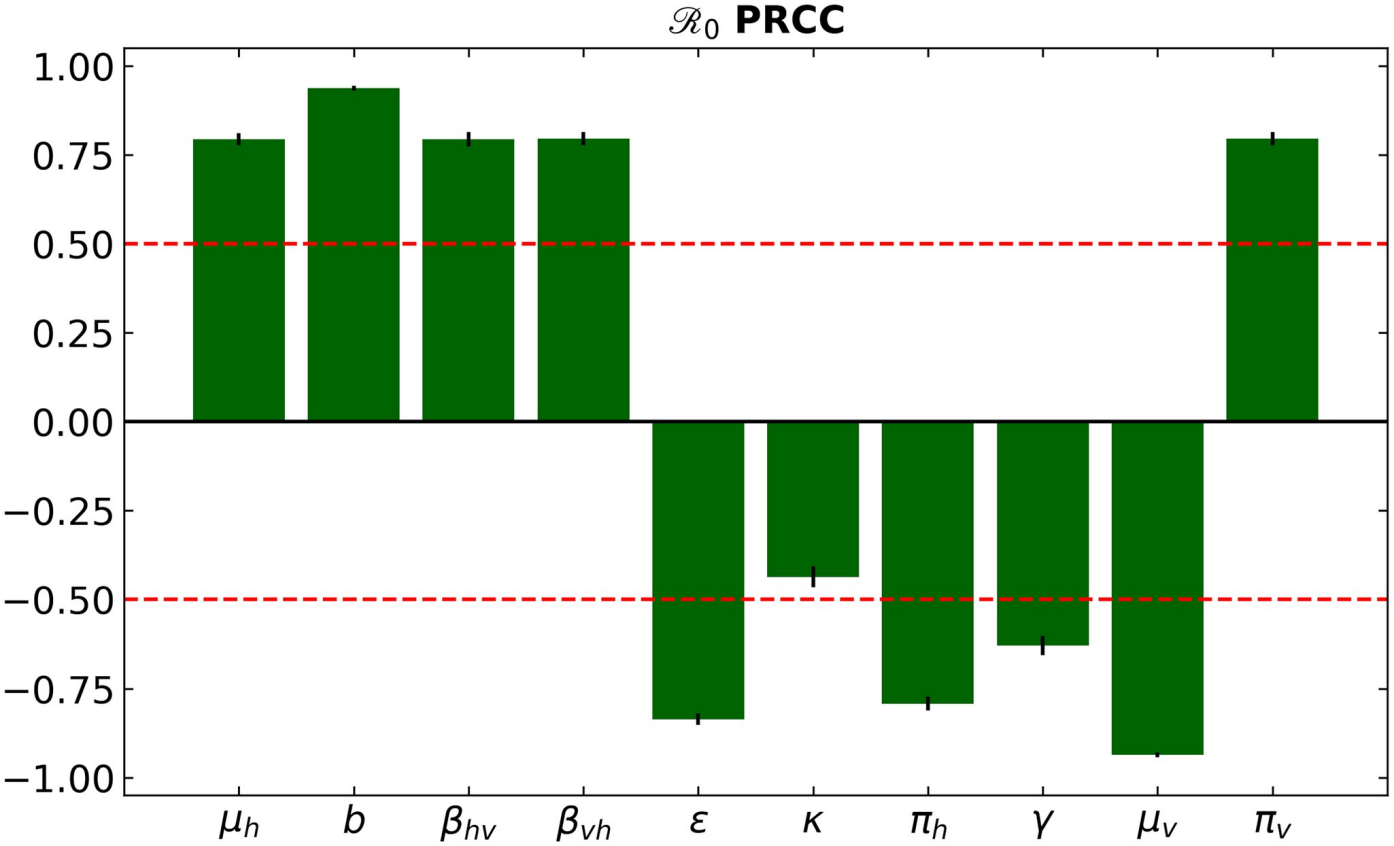
Global sensitivity indices for *R*_0_ against the parameter values in Table 1. This analysis highlights the intercorrelated sensitivities of each of the model parameters. Green bars indicate the mean value of each PRCC, with error bars corresponding to one standard deviation. The red line marks the PRCC values +/- 0.50 and identifies the most influential parameters (greater than 0.50 or less than—0.50).

Fig 9 illustrates the sensitivity analysis results. It indicates that transmission probability, biting rates, vaccine efficacy, Recovery rate for humans rate, and mosquito mortality rates hold the most significant sway over the rise in infections. The first two parameters exhibit negative correlations, while the latter exhibit positive ones. It implies that higher mosquito mortality rates, vaccine, and treatment efficacy contribute to fewer infections. Similarly, reduced biting rates and transmission probabilities lead to fewer dengue cases.

**Fig 10:**
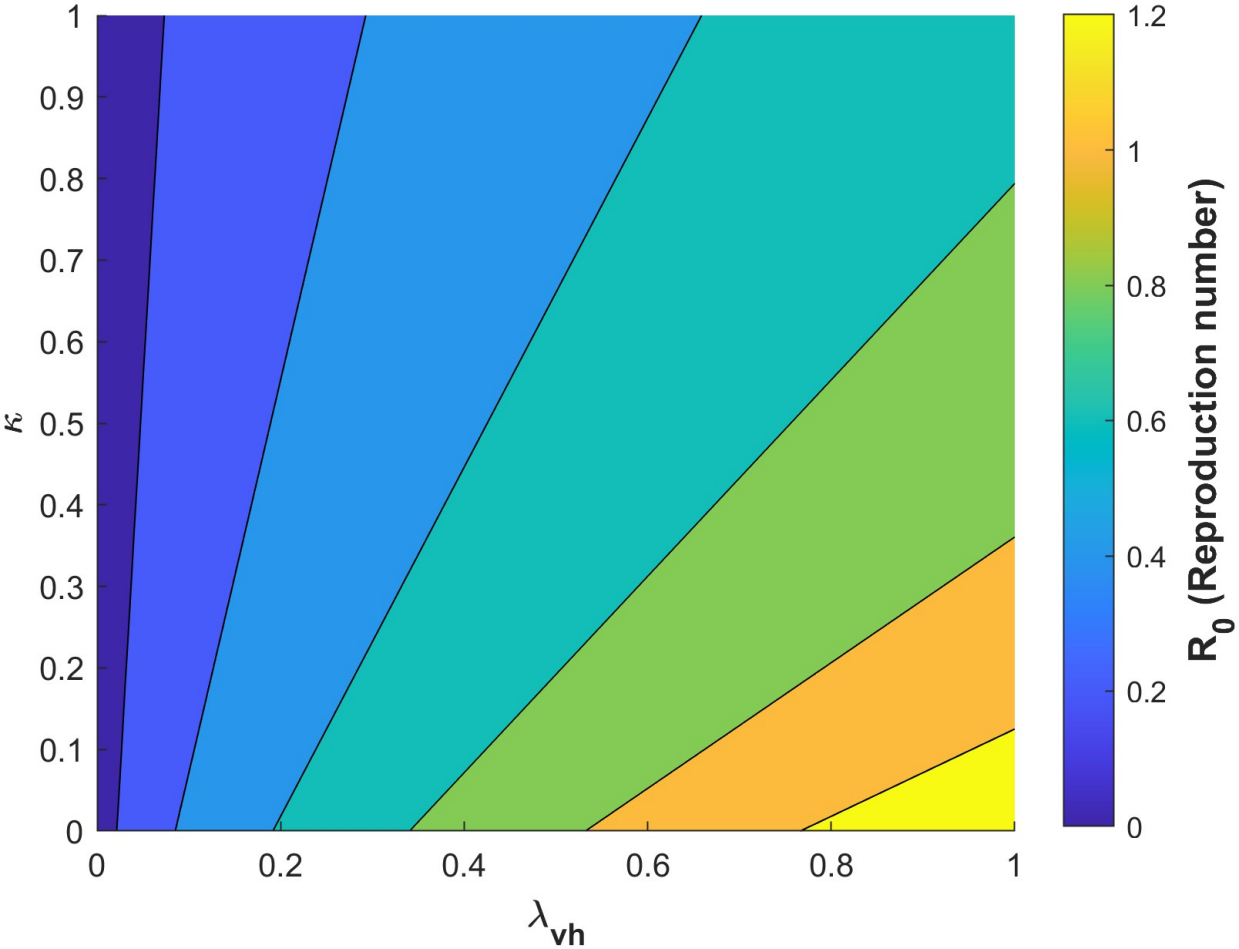
Contour plot of *R*_0_ concerning probability of infection of a susceptible mosquito per bite on an infected human λ_*vh*_ and treatement rate of infectious humans *κ*.

**Fig 11:**
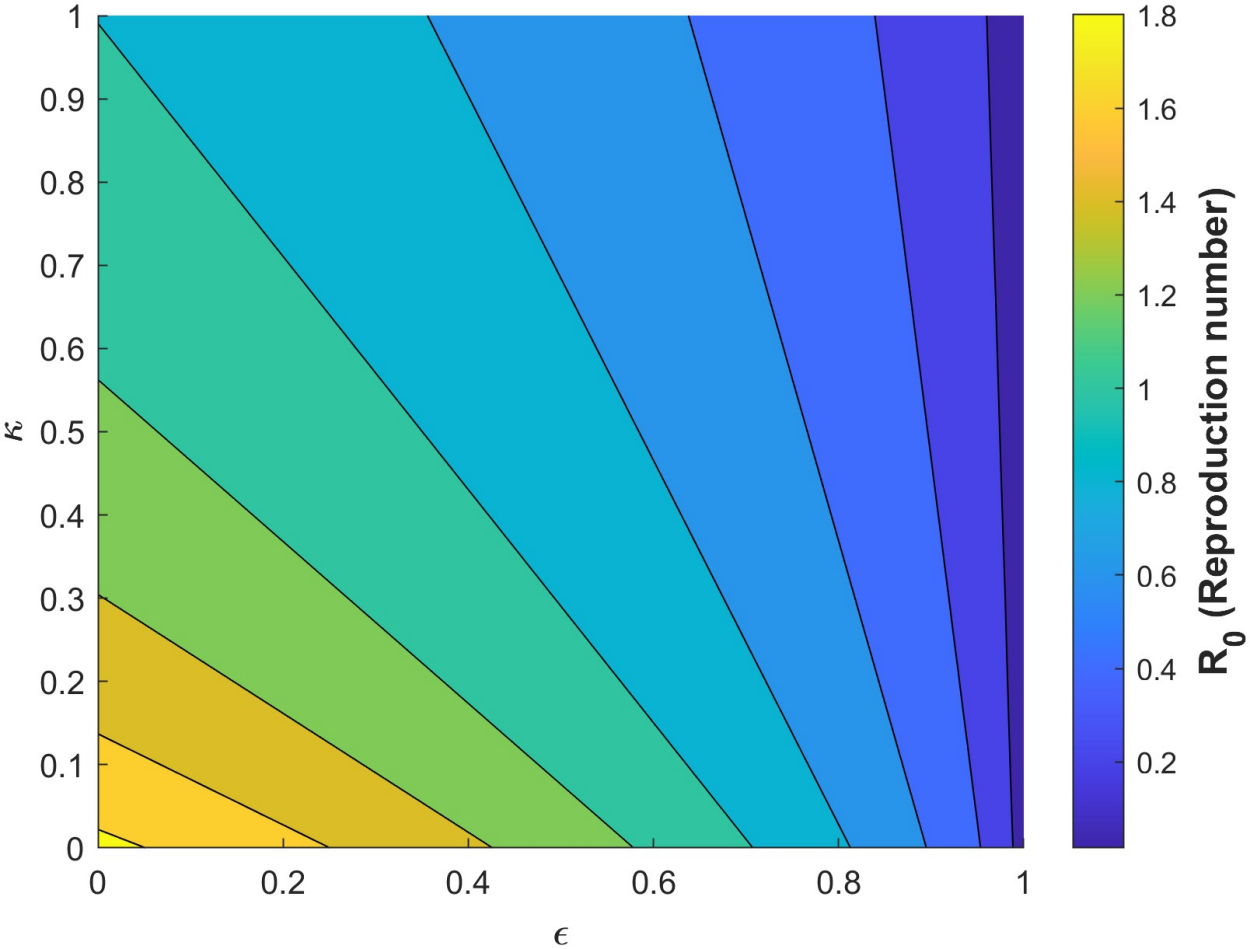
Contour plot of *R*_0_ with respect to vaccine efficacy *ϵ* and treatment rate of infectious humans *κ*.

**Fig 12:**
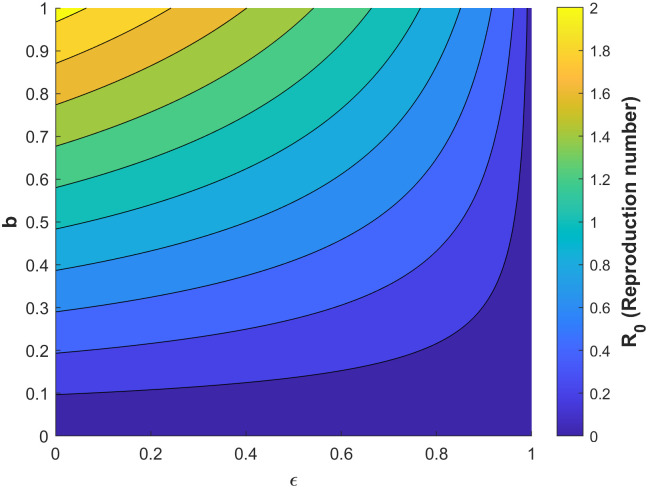
Contour plot of *R*_0_ with respect to vaccine efficacy *ϵ* biting rate *b*.

**Fig 13:**
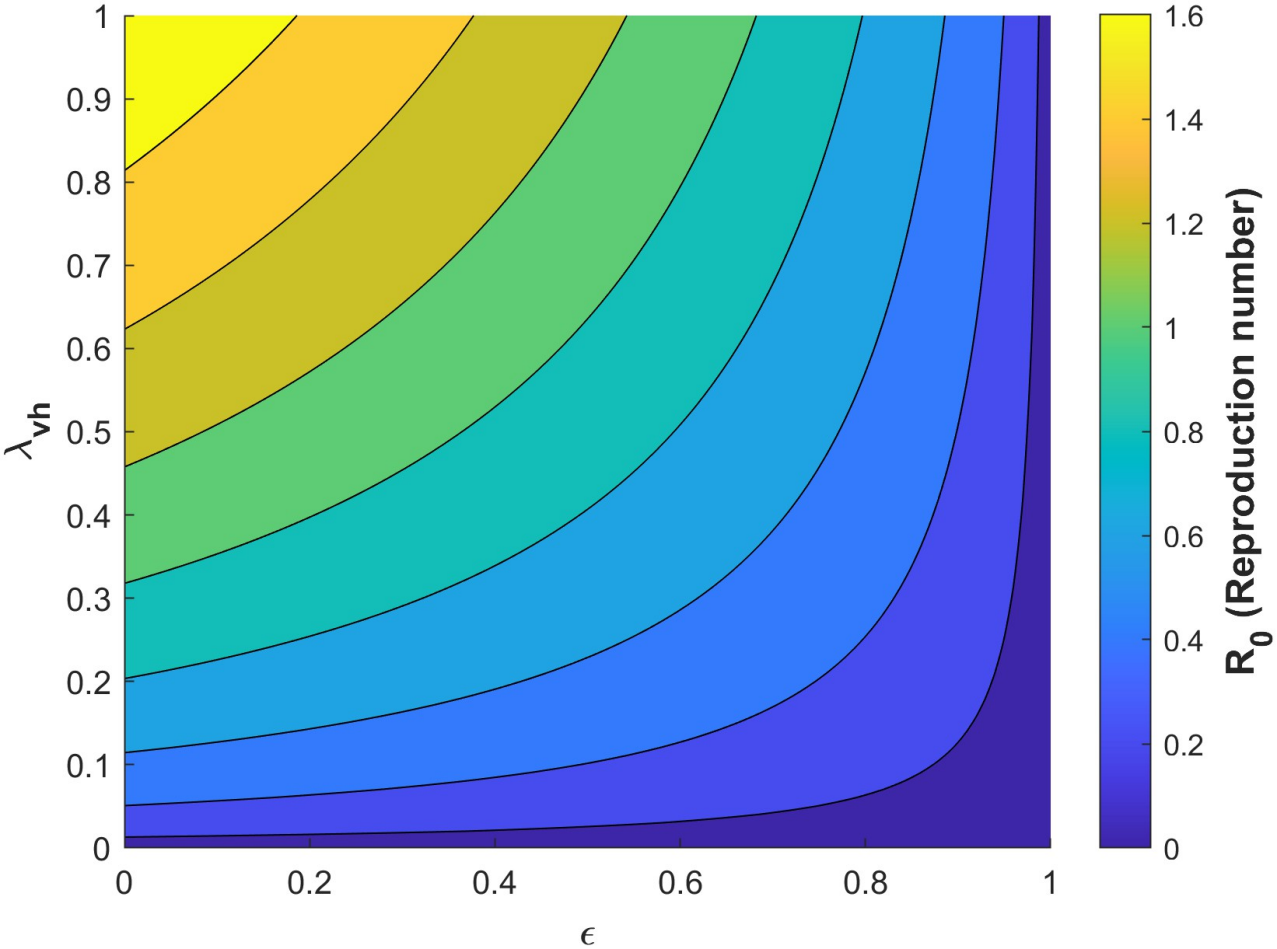
Contour plot of *R*_0_ with respect to vaccine efficacy *ϵ* and treatment rate of infectious humans λ_*vh*_.

**Fig 14:**
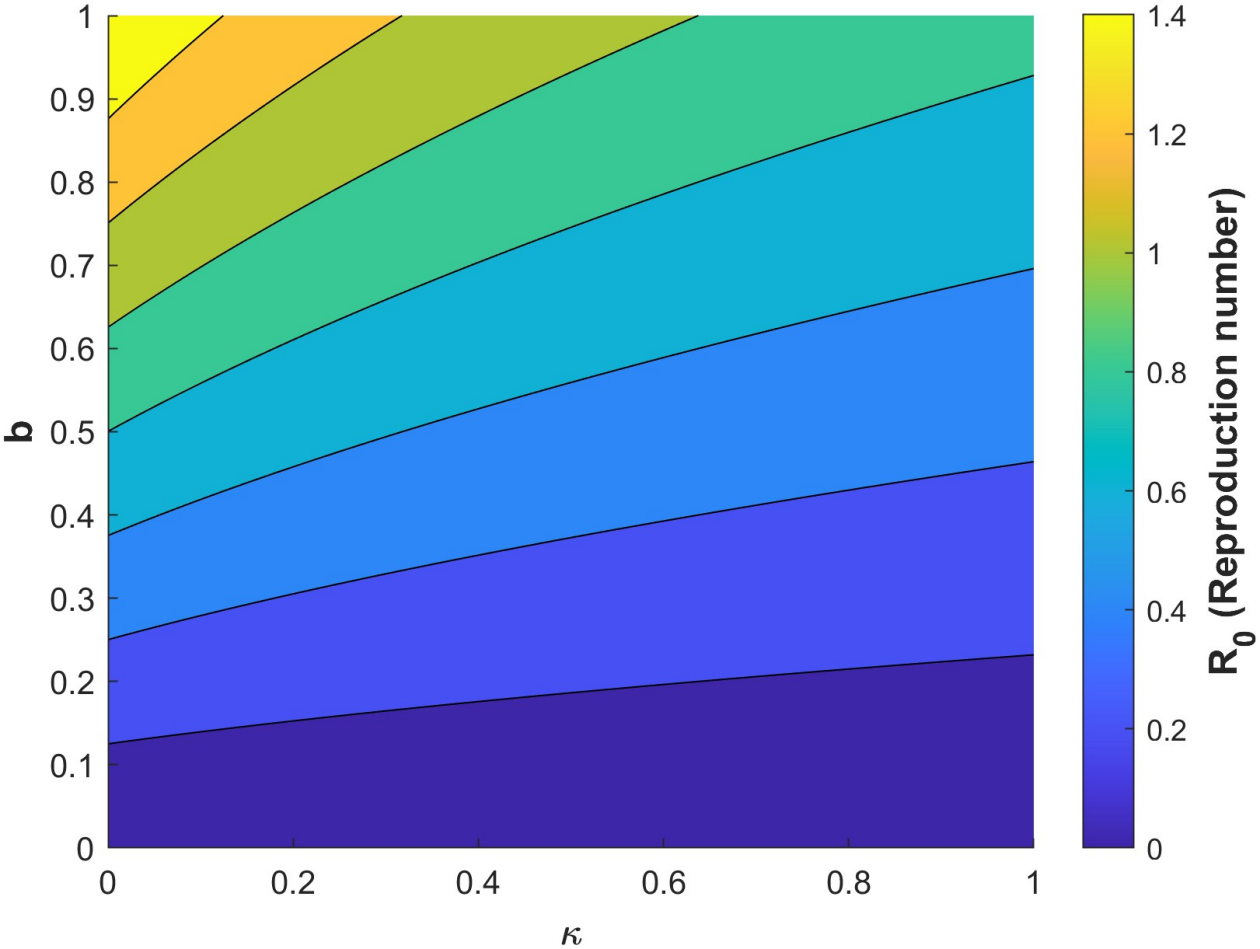
Contour plot of *R*_0_ with respect to biting rate *b* and treatment rate of infectious humans *κ*.

Figs 10–14 confirm the impact of these key parameters on *R*_0_.

## Optimal control model

In the previous section, we investigated the impact of vaccine efficacy and treatment on the dynamics of the infectious disease dengue. In this section, we develop an optimal control problem to address dengue mitigation. We introduce two time-dependent controls, denoted as *u*_1_(*t*) and *u*_2_(*t*) in model (1), to examine how their temporal variations influence dengue dynamics. Eq (14) defines the control problem. To analyze the effect of enhanced vaccine efficacy, we consider the time-dependent control variable *u*_1_(*t*). Similarly, the control variable *u*_2_(*t*) is chosen to assess the improvement in infection treatment. The dengue control model that emerges incorporates these control variables as follows:

$$\begin{array}{c} \frac{dS_{h}}{dt}=\pi_{h}-\lambda_{vh}S_{h}-\left(u_{1}+\mu_{h}\right)S_{h}\\ \frac{dV_{h}}{dt}=u_{1}S_{h}-\left(1-\varepsilon \right)\lambda_{vh}V_{h}-\mu_{h}V_{h}\\ \frac{dI_{h}}{dt}=\lambda_{vh}S_{h}+\left(1-\varepsilon \right)\lambda_{vh}V_{h}-\left(\gamma +\kappa +d+\mu_{h}\right)I_{h}\\ \frac{dT_{h}}{dt}=\kappa I_{h}-\left(u_{2}+d+\mu_{h}\right)T_{h}\\ \frac{dR_{h}}{dt}=\gamma \;I_{h}+u_{2}T_{h}-\mu_{h}R_{h}\\ \frac{dS_{v}}{dt}=\pi_{v}-\lambda_{hv}S_{v}-\mu_{v}S_{v}\\ \frac{dI_{v}}{dt}=\lambda_{hv}S_{v}-\mu_{v}I_{v} \end{array}.$$ (14)

The functional objective for a fixed final time *T* is given below:

$$\begin{array}{c} J\left(u_{1},u_{2}\right)=\int_{0}^{T}(A_{1}I_{h}+A_{2}T_{h}+B_{1}u_{1}^{2}\left(t\right)+B_{2}u_{2}^{2}\left(t\right))dt \end{array}$$ (15)

The principal aim of our observations is to find the optimal control variables $u_{1}^{*}$ and $u_{2}^{*}$ respectively associated with vaccination efficacy and treatment improvement, such that

$$\begin{array}{c} J\left(u_{1}^{*},u_{2}^{*}\right)=\underset{\Omega }{\text{min}}\;J\left(u_{1},u_{2}\right) \end{array}$$

The Lagrangian and Hamiltonian associated with the dengue control system developed Eq (14) are defined as follows

$$H=A_{1}I_{h}+A_{2}T_{h}+B_{1}u_{1}^{2}+B_{2}u_{2}^{2}+\delta_{S_{h}}\frac{dS_{h}}{dt}+\delta_{V_{h}}\frac{dV_{h}}{dt}+\delta_{I_{h}}\frac{dI_{h}}{dt}+\delta_{T_{h}}\frac{dT_{h}}{dt}+\delta_{R_{h}}\frac{dR_{h}}{dt}+\delta_{S_{v}}\frac{dS_{v}}{dt}+\delta_{I_{v}}\frac{dI_{v}}{dt}$$

where $\delta_{S_{h}}$, $\delta_{V_{h}}$, $\delta_{I_{h}}$, $\delta_{T_{h}}$, $\delta_{R_{h}}$, $\delta_{S_{v}}$ and $\delta_{i_{v}}$ are the adjoint variables. The following result presents the adjoint system and control characterization. satisfies

$$\begin{array}{c} \frac{d\delta_{i}}{dt}=-\frac{dH}{d_{i}} \end{array}$$ (16)

where *i* = *S*_*h*_, *V*_*h*_, *I*_*h*_, *T*_*h*_, *R*_*h*_, *S*_*v*_, *I*_*v*_

**Theorem 7** *For the optimal control u*_1_ and *u*_2_ *and the solutions of S*_*h*_, *V*_*h*_, *I*_*h*_, *T*_*h*_, *R*_*h*_, *S*_*v*_ and *I*_*v*_ *of the associated systems that minimizes the objective functional* *J*(*u*_1_, *u*_2_) *over* *U* *there exists adjoint variables* $\delta_{S_{h}}$, $\delta_{V_{h}}$, $\delta_{I_{h}}$, $\delta_{T_{h}}$, $\delta_{R_{h}}$, $\delta_{S_{v}}$, $\delta_{I_{v}}$ *and with transversality condition δ*_*i*_(*T*) = 0 *and*

$$\begin{array}{c} u_{1}=\text{min}\;\{1,\text{max}\;(0,\frac{S_{h}(\delta_{S_{h}}-\delta_{V_{h}})}{2B_{1}})\}\;u_{2}=\text{min}\;\{1,\text{max}\;(0,\frac{T_{h}(\delta_{V_{h}}-\delta_{R_{h}})}{2B_{2}})\} \end{array}$$

**Proof 5** *The adjoint equations are obtained by taking partial derivatives of the Hamiltonian* H *with respect to the associated state variables, so that we obtain*

$$\begin{array}{c} \frac{d\delta_{S_{h}}}{dt}=\delta_{S_{h}}(\frac{b\beta_{vh}I_{v}}{N_{h}}+u_{1}+\mu_{h})-\delta_{V_{h}}u_{1}-\delta_{I_{h}}\frac{b\beta_{vh}I_{v}}{N_{h}}\\ \frac{d\delta_{V_{h}}}{dt}=\delta_{V_{h}}(\left(1-\epsilon \right)\frac{b\beta_{vh}I_{v}}{N_{h}}+\mu_{h})-\delta_{I_{h}}(\left(1-\epsilon \right)\frac{b\beta_{vh}I_{v}}{N_{h}})\\ \frac{d\delta_{I_{h}}}{dt}=-A_{1}+\delta_{I_{h}}\left(\gamma +\kappa +d+\mu_{h}\right)-\delta_{T_{h}}\kappa -\delta_{R_{h}}\gamma +\delta_{S_{v}}\frac{b\beta_{hv}S_{v}}{N_{h}}\left(\delta_{S_{v}}-\delta_{I_{v}}\right)\\ \frac{d\delta_{T_{h}}}{dt}=-A_{2}+\delta_{T_{h}}\left(\mu_{2}+d+\mu_{h}\right)-\delta_{R_{h}}\mu_{h}\\ \frac{d\delta_{R_{h}}}{dt}=\delta_{R_{h}}\mu_{h}\\ \frac{d\delta_{S_{v}}}{dt}=\delta_{S_{v}}\frac{b\beta_{hv}I_{h}}{N_{h}}\left(\delta_{S_{v}}-\delta_{I_{v}}\right)+\delta_{S_{v}}\mu_{v}\\ \frac{d\delta_{I_{v}}}{dt}=\delta_{S_{h}}\frac{b\beta_{vh}}{N_{h}}S_{h}+\delta_{V_{h}}\left(1-\epsilon \right)\frac{b\beta_{vh}}{N_{h}}V_{h}-\delta_{I_{h}}(\frac{b\beta_{vh}}{N_{h}}S_{h}+\left(1-\epsilon \right)\frac{b\beta_{vh}}{N_{h}}V_{h})+\delta_{I_{v}}\mu_{v} \end{array}$$ (17)

*Furthermore, taking the derivative of Hamiltonian with respect to control variables to obtain*

$$\begin{array}{c} \frac{dH}{u_{1}}=2B_{1}u_{1}-\delta_{S_{h}}S_{h}+\delta_{V_{h}}S_{h},\;\frac{dH}{u_{2}}=2B_{1}u_{2}-\delta_{V_{h}}T_{h}+\delta_{R_{h}}T_{h} \end{array}$$ (18)

*Solving the equation for u*_1_ *and* *u*_2_ *to obtain*

$$\begin{array}{c} u_{1}=\frac{S_{h}(\delta_{S_{h}}-\delta_{V_{h}})}{2B_{1}}\;u_{2}=\frac{T_{h}(\delta v_{h}-\delta_{R_{h}})}{2B_{2}} \end{array}$$ (19)

*Using the bounds, we obtain the optimal control characterization as given in* Eq (14). *This completes the proof*.

## Numerical simulations of the optimal controls

In this section, we demonstrate the simulations of our optimal control model using MATLAB. The parameter values remain consistent with those discussed in Section 2. The time interval considered is [0, 100]. To solve the adjoint equations, we explore various strategies to gauge the impact of optimal control on the number of infected individuals. We aim to determine the most effective type of optimal control in reducing the infected population. These scenarios are based on two control intervention approaches: single and paired control variables. The first scenario employs a single control, allowing only one control to be utilized at a time. All controls are applied to the model’s state variables in the second scenario. We delve into each scenario’s impact in greater detail as follows:

Strategy 1: Using only one control at a time

In this scenario, the two control strategies, *u*_1_ (vaccination) and *u*_2_ (treatment), are examined separately to study their impact on disease dynamics.

**Fig 15:**
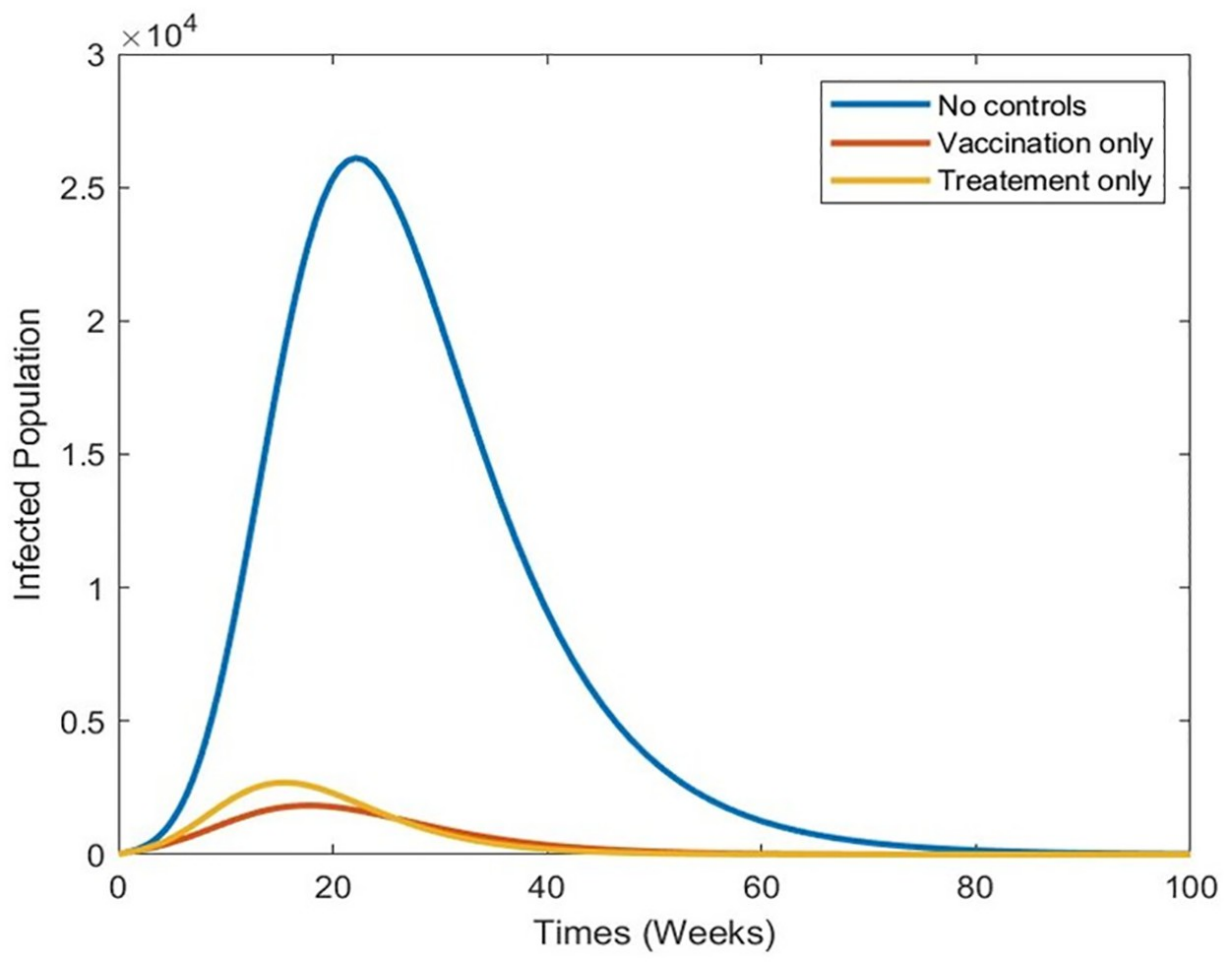
Simulation results of the system of Eq (14) with without controls, vaccination only and treatment only of the infected population.

Fig 15 illustrates the different types of optimal control when applied alone and the corresponding effects on the total number of infected individuals. This figure clearly shows that vaccination is more effective in reducing the incidence of infection.

**Fig 16:**
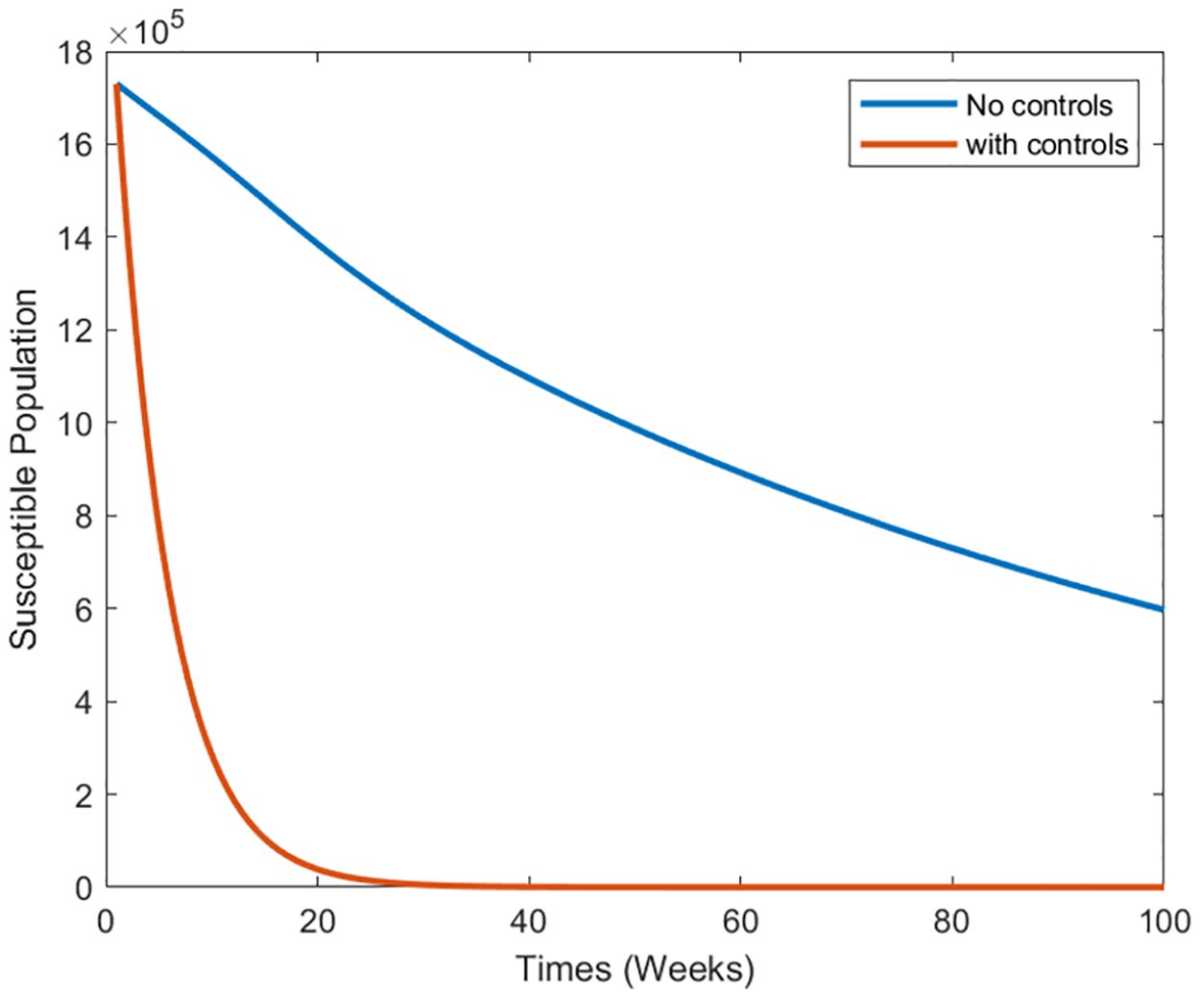
Simulation results of the system of Eq (14) with and without controls of the susceptible population.

**Fig 17:**
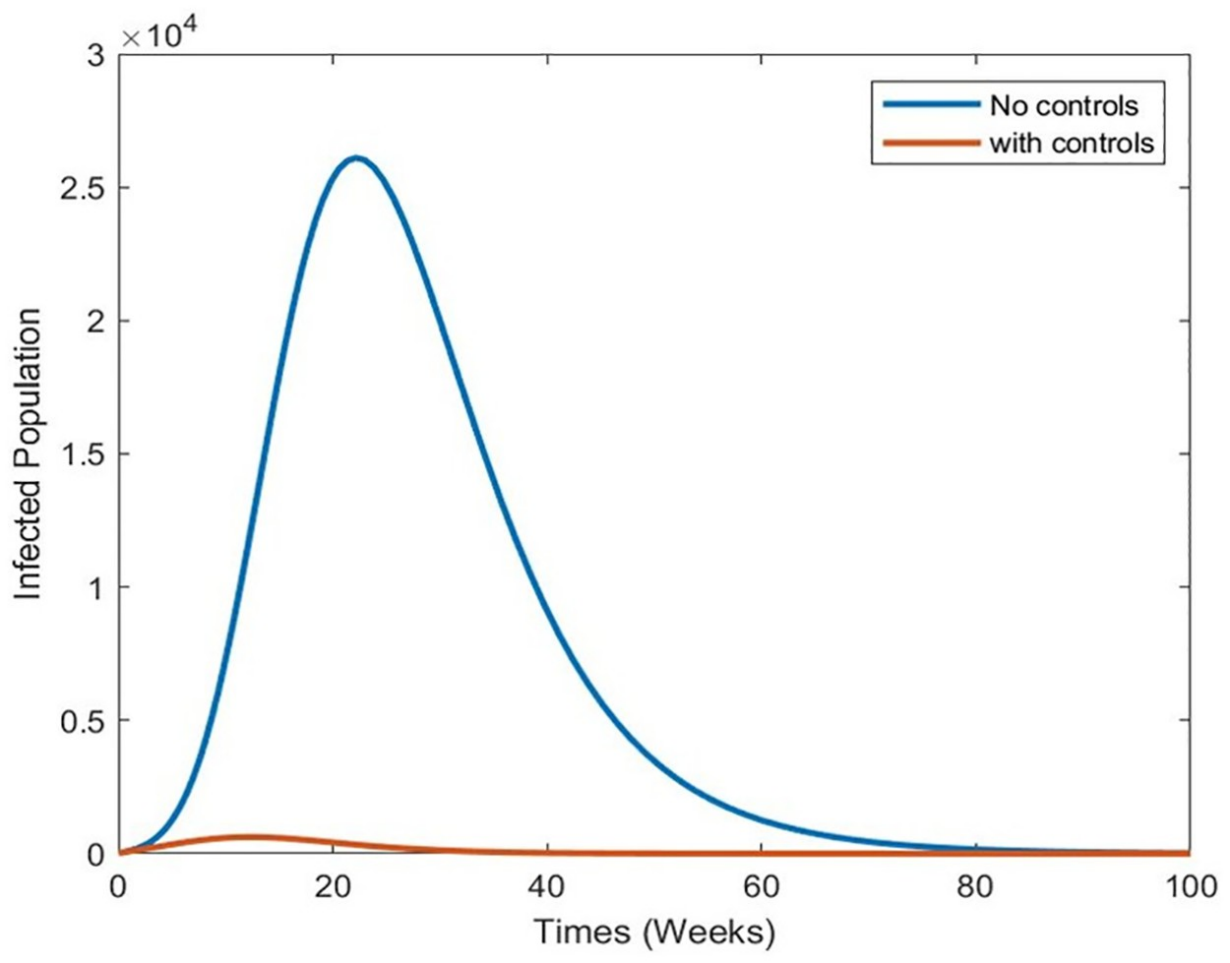
Simulation results of the system of Eq (14) with and without controls of the infected population.

**Fig 18:**
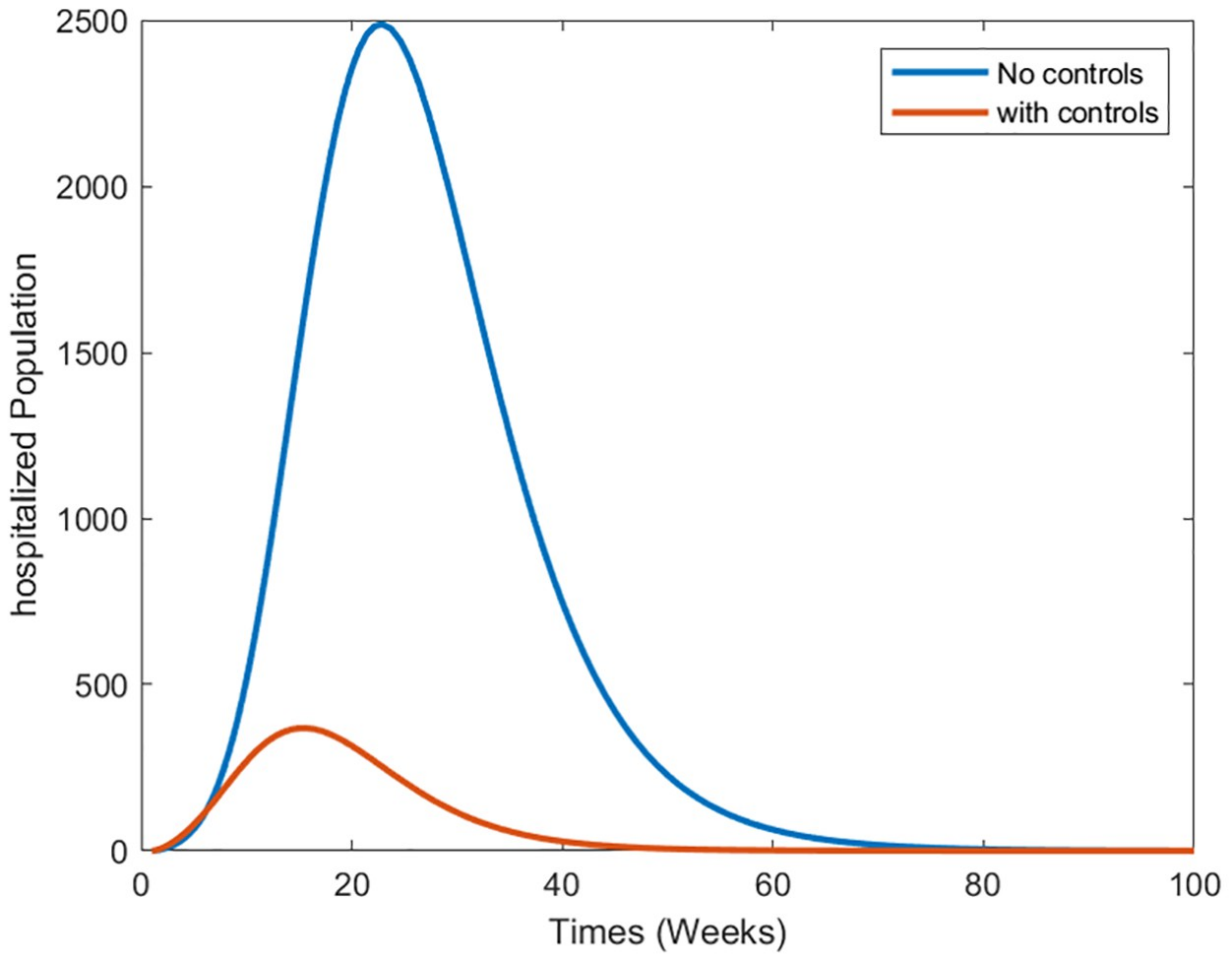
Simulation results of the system of Eq (14) with and without controls of the hospitalized population.

Strategy 2: Implementing all Controls In the second scenario, we examine the collective impact of vaccination and treatment control measures on the dynamics of infectious dengue disease. This exploration aims to elucidate the combined biological implications of simultaneously implementing all control measures for disease incidence. By utilizing the optimal controls within system (14), we present the graphical outcomes in Fig 16 through Fig 18. These figures illustrate the dynamic changes in distinct population compartments under controlled and uncontrolled conditions. Notably, within this dual-control scenario, the susceptible population experiences a rapid decline (Fig 16). Furthermore, the sizes of the infected and hospitalized people (Figs 17 and 18) also witness significant reductions. In summary, simulating this scenario indicates that the simultaneous application of the proposed control measures is more suitable and impactful for reducing infection within a community. This comprehensive approach can potentially protect the population from future disease outbreaks.

## Discussion

This paper introduces a mathematical model for dengue virus disease, considering vaccination and treatment. We calculate the basic reproduction number *R*_0_. The disease-free equilibrium (DFE) is globally asymptotically stable when *R*_0_ < 1 under specific conditions. We estimate the model parameters and validate the model by using the reported dengue infection data in Kaohsiung of the year 2014–2015

Furthermore, treatment performs better than vaccination in reducing dengue transmission when the vaccine’s efficacy is low. However, reducing the disease doesn’t equate to eradicating it. A highly effective vaccine significantly reduces dengue cases if its efficacy is very high. The findings imply that a high vaccine efficacy and vaccination rate make the vaccine alone sufficient. Nonetheless, even with high vaccine effectiveness and vaccination rate, combining it with treatment remains essential. Notably, vaccines with high efficacy aren’t always available. Moreover, the hospitalization of infected patients helps prevent critical cases linked to diseases such as breast cancer.

We conduct a sensitivity analysis to identify the parameter with the most impact on the basic reproduction number *R*_0_. A comprehensive global sensitivity analysis reveals that transmission probability, bite rate, vaccine effectiveness, cure rate, treatment, and mosquito mortality rate significantly affect the increase in infected individuals. Furthermore, the parameters *ϵ* and *κ*, connected to the transmission, hold substantial influence, especially when combined. This synergy is particularly pronounced when both control strategies, vaccination, and treatment, are implemented together.

Moreover, we’ve expanded our model to address the optimal control challenge, incorporating two distinct types of optimal control. We analyze the optimal control problem employing Pontryagin’s maximum principle. We conduct numerical simulations, exploring various combinations of optimal controls. Two scenarios are investigated to minimize infection: strategies involving a single control variable and strategies encompassing all control variables. Notably, using the individual *u*_2_(*t*) (treatment) control strategy alone isn’t effective in reducing infection. However, the simultaneous application of all proposed control measures considerably decreases the overall number of infected individuals.

## Conclusion

This section summarizes the main theoretical results of our study. In this paper, a deterministic compartmental model is developed with a system of seven ODEs that describe vector-host interactions in the presence of vaccination and treatment. After introducing the model, some of its fundamental properties are discussed. In addition, some parameters of the model have been estimated using real data in order to validate it based on the 2014 dengue epidemic in Kaohsiung, Taiwan. To evaluate the impact of different strategies involving the use of vaccination and treatment, an optimal control analysis was established. Theoretical analysis and numerical simulations led the following results.

The disease-free equilibrium (only) is globally stable when the basic reproduction number is less than one. Sensitivity analysis of the models suggests that transmission probability, biting rates, vaccine efficacy, human recovery rates and mosquito mortality rates, among other parameters, are the most important parameters influencing *R*_0_ epidemiological thresholds. By studying the impact of the control measures of vaccination (*u*_1_), and treatment (*u*_2_) on the transmission and spread of the disease in the population, we observe that the combination of the two controls considerably reduces the spread of the disease. Thus, the most effective of the different control strategies analyzed in this work is the strategy combining vaccination and treatment.

In conclusion, our study sheds light on the potential synergies between vaccination and treatment as strategies to mitigate dengue transmission. We have provided valuable insights into how these interventions can work in tandem to curb the spread of the disease. However, it’s important to acknowledge the limitations of our current model, such as the omission of seasonality effects, the impact of age on vaccine efficacy, and the consideration of only one dengue serotype. To enhance the robustness of our findings and provide a more comprehensive understanding of dengue control strategies, future work will delve into incorporating seasonality into the model [27, 28] and exploring the interplay of age [29, 30] and vaccine interventions [11]. Additionally, we have used a single-serotype dengue model that does not take into account the effects of secondary infections. It is therefore advisable to extend this work by considering several dengue serotypes and examining the effects of vaccination and treatment on the dynamics of disease transmission. By continuously refining our model and accounting for these complex factors, we hope to contribute further to developing effective and tailored approaches for managing dengue transmission and ultimately reducing its burden on public health.

## Supporting information

S1 Appendix Presents the value of *c*_1_, *c*_2_ and *c*_3_ in Eq 8. (PDF)
