# Supplementary material for: Interplay between vaccines and treatment for dengue control: An epidemic model
Source: PLoS One. 2024 Jan 25;19(1):e0295025. doi: 10.1371/journal.pone.0295025 (PMC10833586; doi:10.1371/journal.pone.0295025)
Supplement: S1 Appendix — (PDF) [file pone.0295025.s001.pdf]

Appendix. Expressions of the coefficients c_1, c_2, and c_3

$$c_1 = \mu_v^2(k_8 + 3k_{11} + 2k_{12})\mu_v(k_2 + k_3 + 2k_4 + k_5 + 2k_6)$$

$$c_2 = \mu_v(3k_8 + 2k_9 + k_{10} + 3k_{11} + 4k_{12}) - (k_1 + k_2 + k_3 + k_4 + k_5 + k_6)$$

$$c_3 = k_7 + k_8 + 2k_9 + k_{10} + k_{11} + 2k_{12}$$

such that

$$k_1 = A^3 b\lambda_{hv}(1-\epsilon)\pi_h$$

$$k_2 = A^2 b\lambda_{hv}(1-\epsilon)\pi_h(\tau + \mu_h)$$

$$k_3 = A^2 b\lambda_{hv}\pi_h\mu_h$$

$$k_4 = Ab\lambda_{hv}\pi_h\mu_h(\tau + \mu_h)$$

$$k_5 = A^2 b\lambda_{hv}\pi_h\tau(1-\epsilon)$$

$$k_6 = Ab\lambda_{hv}\pi_h\tau(1-\epsilon)(\tau + \mu_h)$$

$$k_7 = \frac{A^3(1-\epsilon)\pi_h(\gamma + \kappa + d + \mu_h)}{\mu_e}$$

$$k_8 = \frac{A(1-\epsilon)\pi_h(\gamma + \kappa + d + \mu_h)(\tau + \mu_h)^2}{\mu_h}$$

$$k_9 = \frac{2A^2(1-\epsilon)\pi_h(\gamma + \kappa + d + \mu_h)(\tau + \mu_h)}{\mu_h}$$

$$k_{10} = A^2\pi_h(\gamma + \kappa + d + \mu_h)$$

$$k_{11} = A\pi_h(\gamma + \kappa + d + \mu_h)(\tau + \mu_h)^2$$

$$k_{12} = 2A\pi_h(\tau + \mu_h)(\gamma + \kappa + d + \mu_h)$$

and

$$A = \frac{b\lambda_{vh}\pi_v\mu_h}{\pi_h\mu_v}$$
